# Fitness Trade-Offs in Phage Cocktail-Resistant Salmonella enterica Serovar Enteritidis Results in Increased Antibiotic Susceptibility and Reduced Virulence

**DOI:** 10.1128/spectrum.02914-22

**Published:** 2022-09-27

**Authors:** Dongyang Gao, Hongyue Ji, Linkang Wang, Xinxin Li, Dayue Hu, Junna Zhao, Shuang Wang, Pan Tao, Xiangmin Li, Ping Qian

**Affiliations:** a State Key Laboratory of Agricultural Microbiology, Huazhong Agricultural University, Wuhan, Hubei, People’s Republic of China; b College of Veterinary Medicine, Huazhong Agricultural University, Wuhan, Hubei, People’s Republic of China; c Key Laboratory of Preventive Veterinary Medicine in Hubei Province, the Cooperative Innovation Center for Sustainable Pig Production, Wuhan, Hubei, People’s Republic of China; Huazhong University of Science and Technology

**Keywords:** *Salmonella* Enteritidis, phage therapy, phage cocktails, phage receptors, fitness costs

## Abstract

The rapid emergence of phage-resistant bacterial mutants is a major challenge for phage therapy. Phage cocktails have been considered one approach to mitigate this issue. However, the synergistic effect of randomly selected phages in the cocktails is ambiguous. Here, we rationally designed a phage cocktail consisting of four phages that utilize the lipopolysaccharide (LPS) O antigen, the LPS outer core, the LPS inner core, and the outer membrane proteins BtuB and TolC on the Salmonella enterica serovar Enteritidis cell surface as receptors. We demonstrated that the four-phage cocktail could significantly delay the emergence of phage-resistant bacterial mutants compared to the single phage. To investigate the fitness costs associated with phage resistance, we characterized a total of 80 bacterial mutants resistant to a single phage or the four-phage cocktail. We observed that mutants resistant to the four-phage cocktail were more sensitive to several antibiotics than the single-phage-resistant mutants. In addition, all mutants resistant to the four-phage cocktail had significantly reduced virulence compared to wild-type strains. Our mouse model of Salmonella Enteritidis infection also indicated that the four-phage cocktail exhibited an enhanced therapeutic effect. Together, our work demonstrates an efficient strategy to design phage cocktails by combining phages with different bacterial receptors, which can steer the evolution of phage-resistant strains toward clinically exploitable phenotypes.

**IMPORTANCE** The selection pressure of phage promotes bacterial mutation, which results in a fitness cost. Such fitness trade-offs are related to the host receptor of the phage; therefore, we can utilize knowledge of bacterial receptors used by phages as a criterion for designing phage cocktails. Here, we evaluated the efficacy of a phage cocktail made up of phages that target four different receptors on Salmonella Enteritidis through *in vivo* and *in vitro* experiments. Importantly, we found that pressure from phage cocktails with different receptors can drive phage-resistant bacterial mutants to evolve in a direction that entails more severe fitness costs, resulting in reduced virulence and increased susceptibility to antibiotics. These findings suggest that phage cocktail therapy using combinations of phages targeting different important receptors (e.g., LPS or the efflux pump AcrAB-TolC) on the host surface can steer the host bacteria toward more detrimental surface mutations than single-phage therapy, resulting in more favorable therapeutic outcomes.

## INTRODUCTION

Salmonella enterica serovar Enteritidis is one of the most frequently isolated nontyphoidal Salmonella serotypes that cause illnesses in humans, including self-limiting gastroenteritis, severe fever, bacteremia, meningitis, and other local infections, which occur in both developing and developed countries (1, 2). Most human cases of infection can be effectively treated with antibiotics. However, antibiotic resistance in Salmonella, especially multidrug-resistant (MDR) virulent strains, compromises the successful control of human salmonellosis and poses the most significant threat to global public health (3, 4). Thus, the emergence of MDR bacterial pathogens has led to the exploration of alternative antibiotics and therapeutic strategies (5).

Bacteriophage therapy, i.e., the use of lytic phages to destroy pathogenic bacteria, is a promising strategy in the fight against antimicrobial resistance (6–8). In contrast to antibiotics, phages have a unique ability to self-proliferate at the site of infection with high host specificity and do not infect the native microflora of the host organism (5, 9). However, a major concern regarding the use of phage therapy is that bacteria can develop phage-resistant mutants, which greatly limits the therapeutic potential of phages (10, 11). Previous studies indicated that phage cocktails can overcome this shortcoming (12–16). However, little is known about the advantages of phage cocktails containing different types of phages that bind host bacterial cells through different receptors.

The first step of phage infection is adsorption to specific receptors on the surface of bacteria, including outer membrane proteins (OMPs), sugar residues in the O antigen or lipopolysaccharide (LPS) core, teichoic acid, polysaccharides of the capsule or slime layer, flagella, and fimbriae (17). Bacteria typically evolve phage resistance by deleting or inactivating phage-specific receptors. The binding of phages to its receptors exerts selective pressure on bacteria, which alters the expression of the receptors, thereby preventing phage infection (11). The selection pressure of phage promotes bacterial mutation, which results in a fitness cost and trade-offs in return (18–20). If phage receptors are associated with virulence factors or antibiotic resistance mechanisms in the target bacteria, such fitness trade-offs might reduce the virulence or antibiotic resistance of the pathogenic bacteria (5). For example, some phages utilize the antibiotic efflux pump as a receptor, and the phage-resistant mutants with impaired efflux pumps have increased susceptibility to several classes of antibiotic drugs (21, 22). Phages utilize components of LPS as receptors, and the phage-resistant mutants resulting from alteration of LPS generally have reduced fitness and virulence (23). Such interactions demonstrate that receptor-based steering of phage resistance could facilitate phage therapy. In particular, we can optimize the phage cocktail by including multiple phages that utilize different receptors on bacteria to avoid cross-resistance (24, 25).

In this study, we aimed to develop an optimal phage cocktail utilizing bacterial receptors used by phages. We evaluated the efficacy of a phage cocktail made up of phages that target four different receptors on S. Enteritidis through *in vivo* and *in vitro* experiments. Furthermore, we investigated the trade-offs exhibited by phage-resistant mutants of S. Enteritidis. We found that the increased cost of phage resistance is related to the evolutionary pressure on host bacteria; i.e., the pressure of phage cocktails with different receptors can drive the evolution of phage-resistant bacterial mutants in a direction that entails more severe fitness costs. Therefore, we propose that phage cocktails can be rationally designed to reduce the likelihood of bacteria evolving resistance and increase the fitness cost of bacterial resistance evolution, thereby promoting the therapeutic efficacy of phages.

## RESULTS

### Selection of phages based on host receptors.

**FIG 1:**
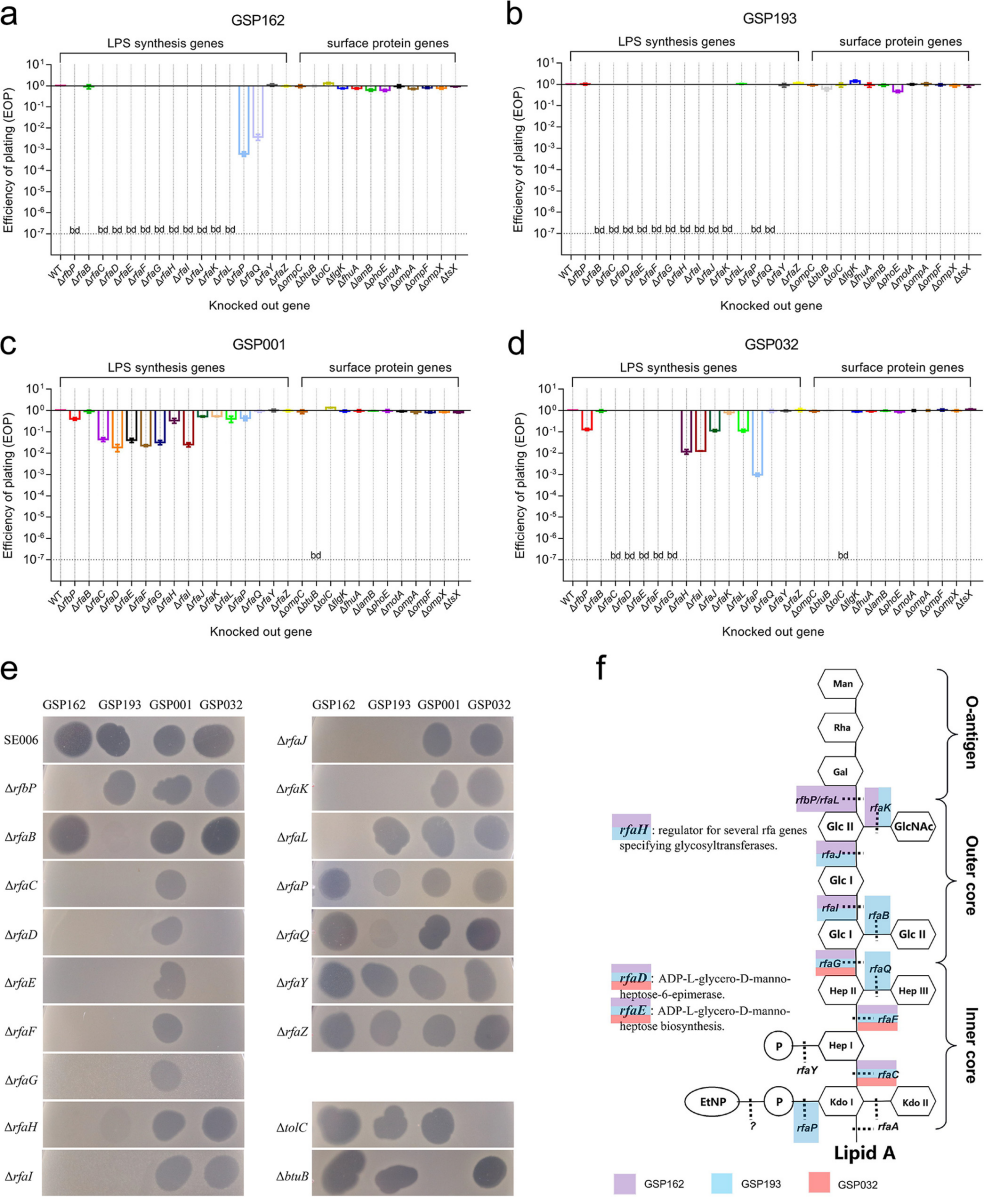
Screening of receptors utilized by phages GSP162, GSP193, GSP001, and GSP032 for infecting S. Enteritidis. (a to d) Receptor screen of phage GSP162 (a), GSP193 (b), GSP001 (c), and GSP032 (d) by determining the EOP (phage titer of knockout strain/phage titer of wild-type SE006 strain) of strains with knockouts of LPS- and OMP-related genes. “bd” on the dotted line indicates that the values were below the limit of detection. (e) Phage GSP162, GSP193, GSP001, and GSP032 stock (about 10^9^ to 10^10^ PFU/mL) were spotted on lawns of S. Enteritidis SE006 knockout strains. Phage stock was spotted on Δ*rfaH* (phage GSP162 and GSP193), Δ*rfaP* (phage GSP193), and Δ*rfaQ* (phage GSP193) lawns, which produced only one confluent lysis zone, not individual plaque lysis. (f) Schematic structure of LPS, showing that different genes required for LPS synthesis affect the plaque formation (EOP) of phage GSP162, GSP193, and GSP032 (regions highlighted in red, blue, and purple). The structure of LPS is that for Salmonella enterica serovar Typhimurium, modified from references 62–64, and 65.

To select phages that utilize different receptors for infection, we constructed a mutant library of S. Enteritidis comprising bacteria deficient in LPS synthesis and OMP-related genes (see Table S3 in the supplemental material). Two hundred sixteen phages were then screened for the ability to infect mutant library strains of S. Enteritidis lacking candidate phage receptors. Four phages that utilize different receptors were then selected and validated by efficiency of plating (EOP) in various gene knockout strains (Fig. 1a to d). Phage stock spotted on Δ*rfaH* (phage GSP162, and GSP193), Δ*rfaP* (phage GSP193), and Δ*rfaQ* (phage GSP193) lawns formed a confluent zone of lysis (Fig. 1e), but serial dilutions of phage spotted on the same lawn could not generate plaques, so EOP was calculated as below the limit of detection.

**FIG 2:**
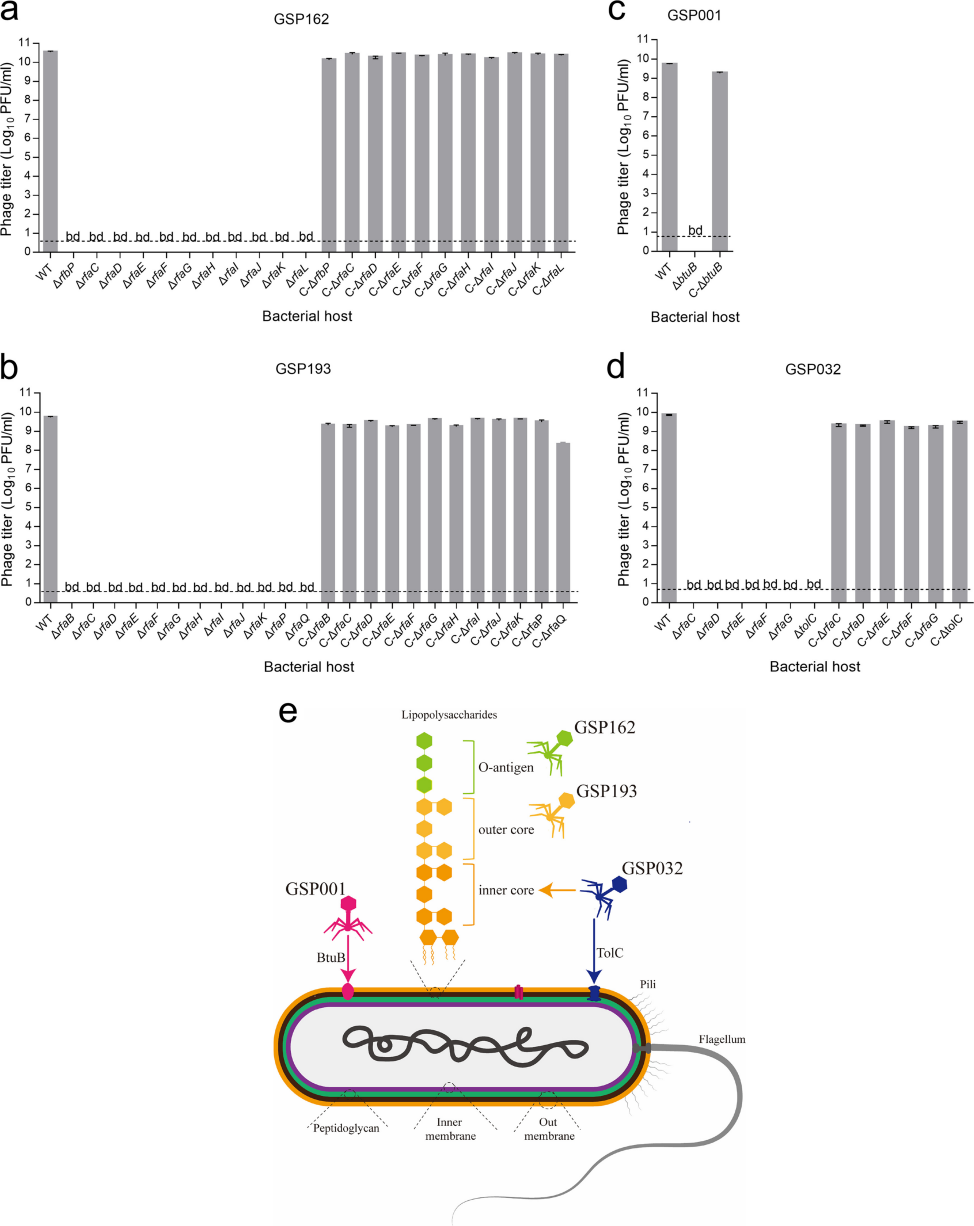
Determination of S. Enteritidis receptor sites for phage recognition. (a to d) Gene knockout strains were complemented with receptor genes of phage GSP162 (a), GSP193 (b), GSP001 (c), and GSP032 (d), completely restoring the plaque-forming ability. bd, below the limit of detection. (e) Proposed model for phage recognition of the receptor of S. Enteritidis SE006.

No plaques were produced when S. Enteritidis mutants with deletions of LPS synthesis genes were infected by phages GPS162 and GPS193, whereas knockout of OMPs did not restrict infection by phages GPS162 and GPS193 (Fig. 1a and b). The OMP *btuB* gene was required for phage GSP001 plaque formation, whereas knockout of LPS synthesis genes had only a minor effect on EOP (Fig. 1c). Phage GSP032 could not propagate on S. Enteritidis mutants which lack LPS synthesis genes or the OMP gene *tolC*, indicating that both LPS and TolC are required for infection (Fig. 1d). We confirmed that these genes were necessary to influence plaque formation using genetic complementation tests (Fig. 2a to d). In addition, the ability of phages GSP162, GSP193, GSP001, and GSP032 to lyse knockout strains in liquid medium was also tested (see Fig. S1).

Deletion of genes responsible for various steps in LPS synthesis would lead to differential truncations of LPS; this would result in disruption of the O antigen or core region. From the known function of LPS synthesis genes that affected plaque formation by various phages, it could be determined that phages GSP162, GSP193, and GSP032 likely target different regions of LPS (Fig. 1f). GSP162 mainly targets the O antigen (requires *rfbP* and *rfaL*), GSP193 mainly targets the outer-core region of LPS (requires *rfaB*, *rfaG*, *rfaH*, *rfaI*, *rfaJ*, and *rfaK*), and GSP032 mainly targets the inner-core region of LPS (requires *rfaC*, *rfaD*, *rfbE*, and *rfaF*) (Fig. 1f). The receptors that were determined to be necessary for these four phages to infect S. Enteritidis SE006 are illustrated in Fig. 2e.

### Host range of four selected phages.

The host ranges of the four selected phages were determined using different Salmonella, Escherichia coli, Listeria monocytogenes, Staphylococcus aureus, and Klebsiella pneumoniae strains. Plaque results showed that phage GSP193 could lyse all 48 tested Salmonella strains belonging to 19 different Salmonella serovars. Phages GSP162, GSP001, and GSP032 were able to successfully infect 36, 42, and 31, respectively, of the tested Salmonella strains. In addition, GSP032 was also able to lyse E. coli strains DH5α and BL21. EOP values ranged from 0.0001 to less than 1.5 (Fig. S3).

### Infection dynamics of phages *in vitro*.

**FIG 3:**
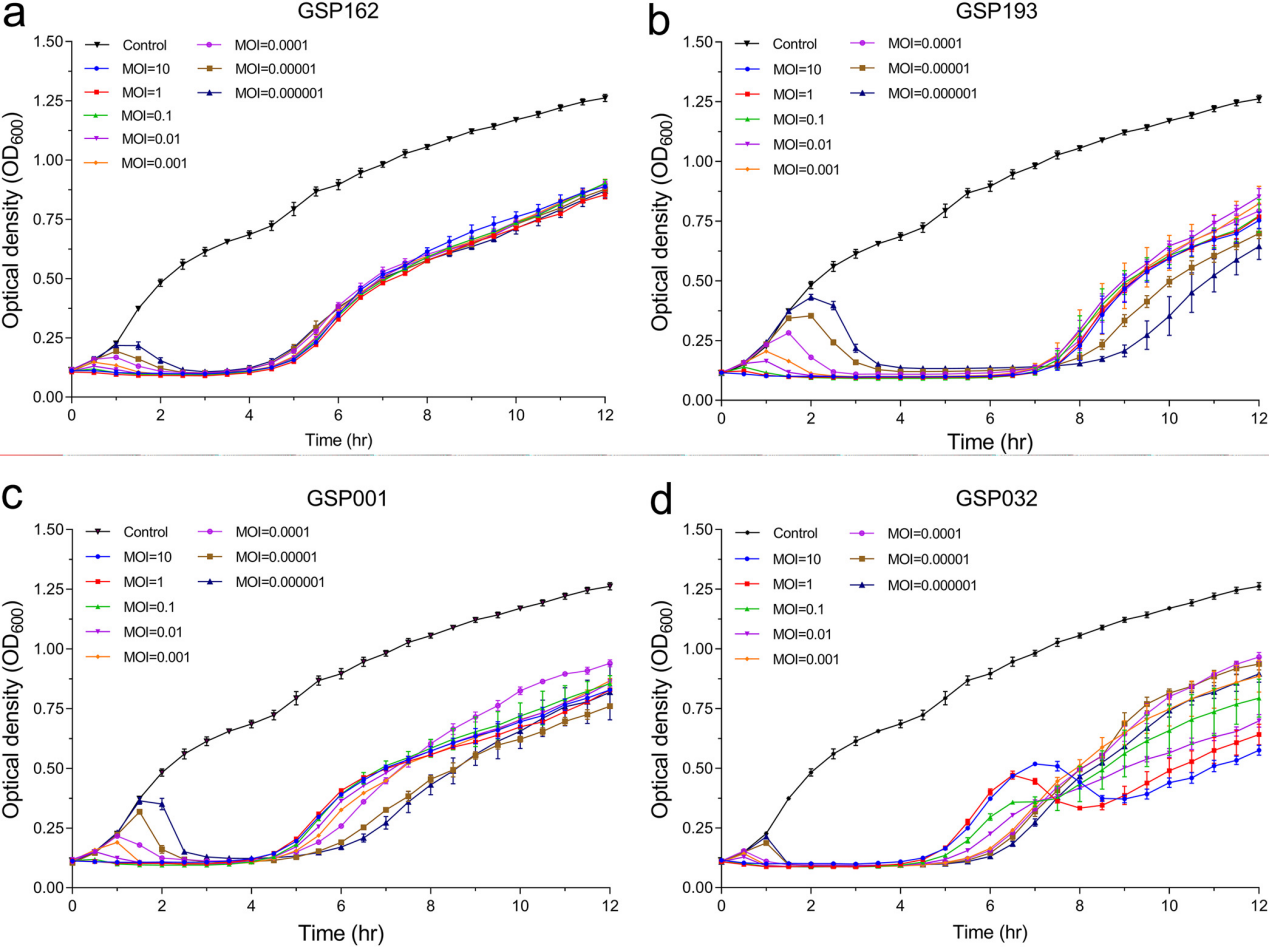
Lytic activity of phages GSP162, GSP193, GSP001, and GSP032 in LB medium. (a to d) Lytic activity of phage GSP162 (a), GSP193 (b), GSP001 (c), and GSP032 (d) against S. Enteritidis SE006 in LB medium at MOI of 10, 1, 0.1, 0.01, 0.001, 0.0001, 0.00001, and 0.000001 at 37°C. Data are means and SD from three independent experiments.

**FIG 4:**
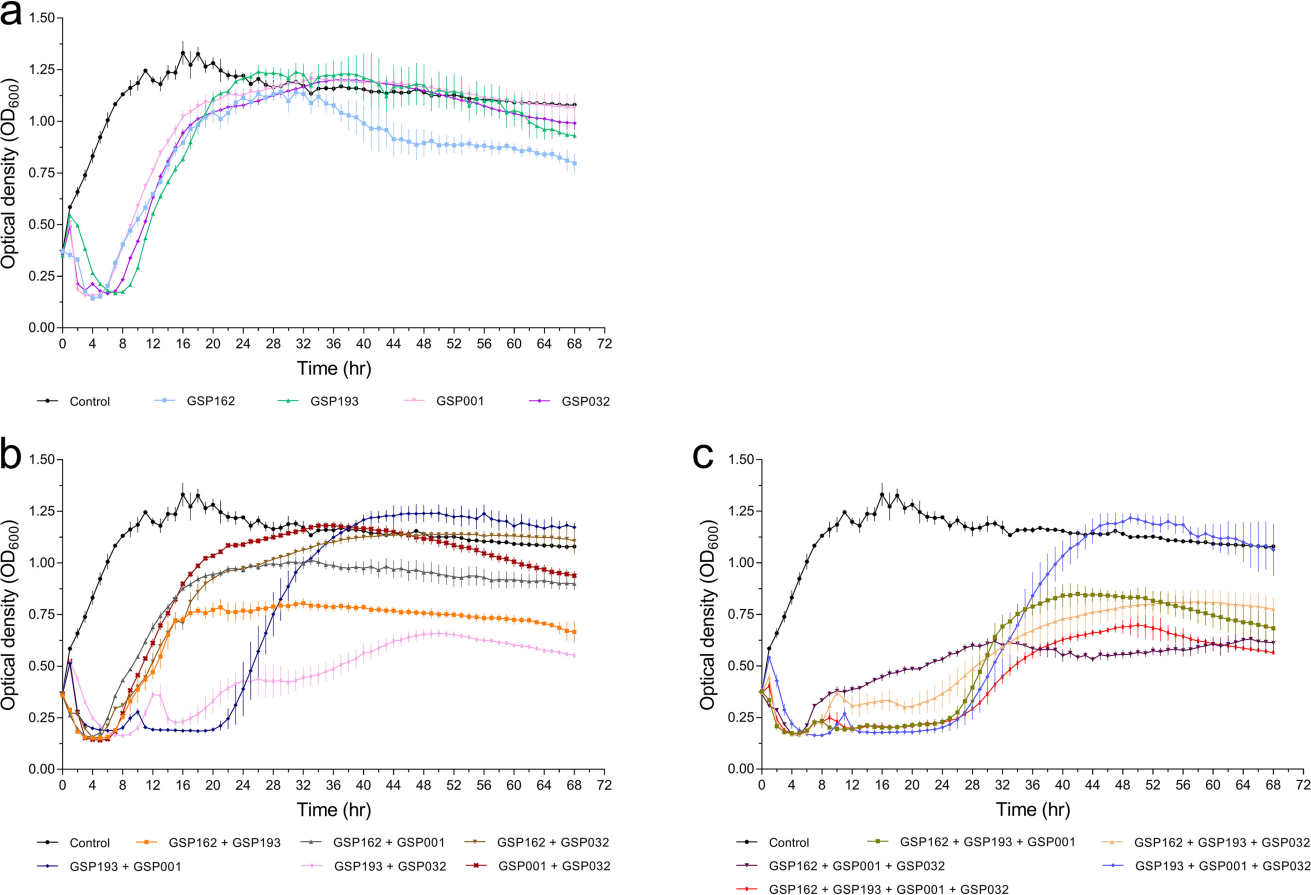
*In vitro* killing of S. Enteritidis SE006 by single phages and phage cocktails. (a) Single phages. (b) Cocktails with two phages. (c) Cocktails with three phages and four phages. Strain SE006 was incubated until early exponential phase (OD_600_ = 0.3 to 0.4) in LB medium, at which point different phage combinations (Table S5) were added to the culture (MOI = 1), and the growth curve was continuously monitored until 68 h. Data are means and SD from three independent experiments.

The lytic activity of four phages *in vitro* was tested by determining the growth kinetics of the S. Enteritidis SE006 strain infected by different phages at different multiplicities of infection (MOI) (MOI = 0.000001, 0.00001, 0.0001, 0.001, 0.01, 0.1, 1, and 10). Each phage successfully delayed the growth of the host bacteria, despite mild differences in inhibition patterns among the four phages (Fig. 3a to d). The emergence of phage-resistant mutants was observed shortly after treatment with only a single phage. We then sought to determine the efficacy of phage combinations for inhibiting bacterial growth and limiting resistance mutations. Therefore, we generated phage communities containing every possible combination (single, double, triple, or quadruple phages) of the four selected phages (Table S5). Bacterial killing curves after infection with the phage communities demonstrated that phage combinations had a distinct advantage in inhibiting the growth of host bacteria compared to a single phage (Fig. 4). Importantly, we observed that the combination of all four phages could continually inhibit bacterial growth for at least 24 h, and the bacterial cell density (optical density at 600 nm [OD_600_]) remained less than 0.75 until 68 h. These results demonstrated that the four-phage combination was effective in delaying the emergence of phage-resistant mutants.

### Cross-resistance of the resistant mutants to the four phages.

**FIG 5:**
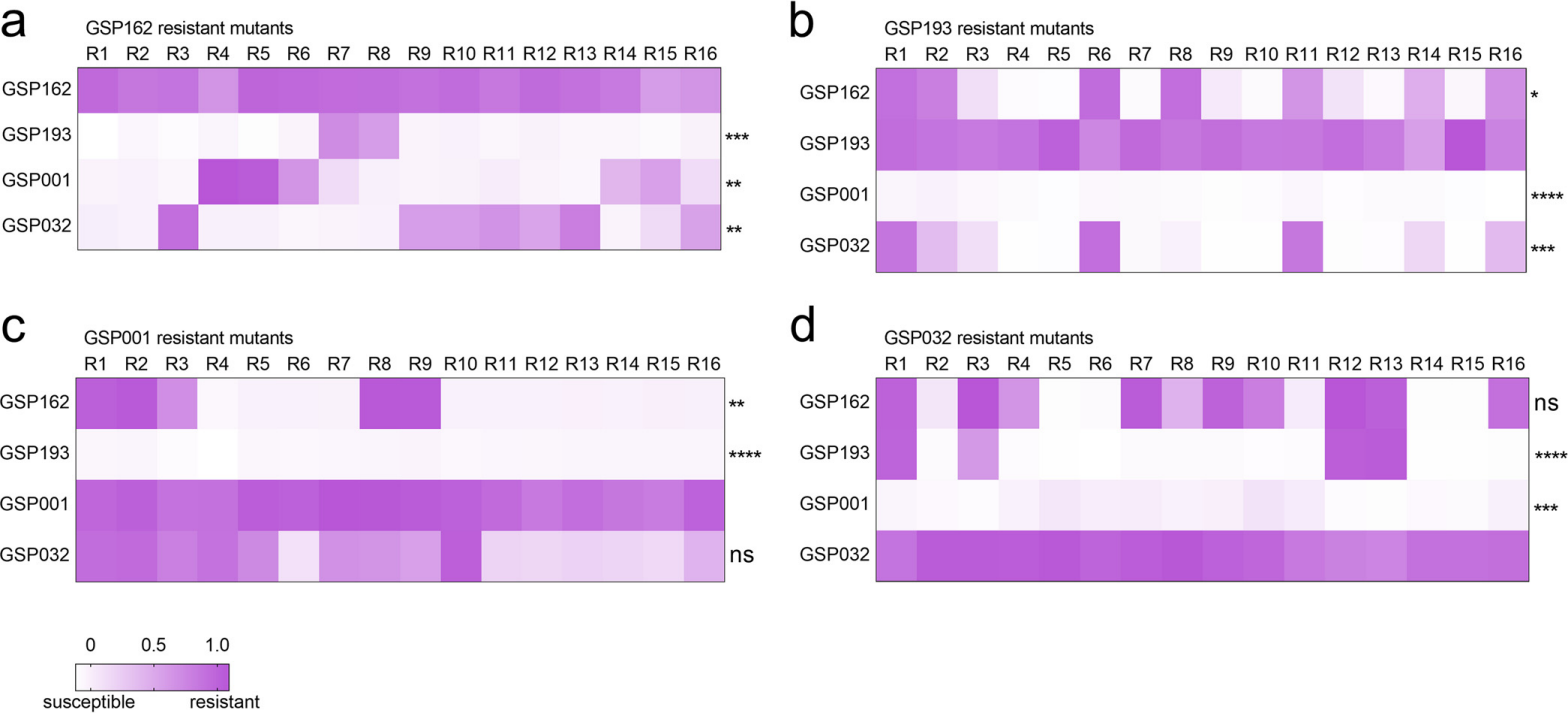
Cross-resistance analysis of S. Enteritidis phage-resistant mutants. (a to d) Cross-resistance of S. Enteritidis mutants to phages GSP162 (a), GSP193 (b), GSP001 (c), and GSP032 (d). Cross-resistance is presented as RBG values. RBG was calculated by comparing the absorbance readings (OD_600_) after 4 h of bacterial growth in the presence and absence of phage (growth curves of the phage-resistant strains used to calculate the RBG are provided in Fig. S5). Each column represents one of 16 mutants resistant to a single phage, infected with a different phage. Each row represents a phage infecting a different phage-resistant mutant. The shading reflects the extent of resistance, ranging from 1 (purple; full resistance) to 0 (white; full susceptibility). Statistical comparisons were performed relative to the control (GSP162-, GSP193-, GSP001-, and GSP032-resistant mutants) using the Kruskal-Wallis test with Dunn’s *post hoc* multiple-comparison test (ns, not significant; *, *P < *0.05; **, *P < *0.01; ***, *P < *0.001; ****, *P < *0.0001).

To determine the extent of cross-resistance of the resistant mutants to the four phages, we tested 64 phage-resistant S. Enteritidis mutants selected against each of four phages (i.e., 16 mutants with resistance against each phage were selected). We then measured growth curves of each S. Enteritidis mutant in the presence and absence of phages (Fig. S5). The extent of cross-resistance was then determined by the relative bacterial growth (RBG) value (Fig. 5). The phage GSP162-resistant SE006 strain was more sensitive to phage GSP193 (*P < *0.001) than phages GSP001 (*P < *0.01) and GSP032 (*P < *0.01), while phage GSP193-resistant mutants were more susceptible to phage GSP001 (*P < *0.0001). Meanwhile, phage GSP001-resistant mutants could also be infected by phage GSP193 (*P < *0.0001). Unexpectedly, phage GSP032 could hardly infect phage GSP001-resistant mutants, but phage GSP001 could infect phage GSP032-resistant mutants (*P < *0.001). In summary, we did not find that acquired bacterial resistance to one phage resulted in resistance to all other phages or complete reciprocal (i.e., symmetric) cross-resistance pairwise among phages. These results suggested that the four phages targeting different receptors have the potential to kill bacteria synergistically.

### Fitness trade-offs of phage-resistant mutants.

**FIG 6:**
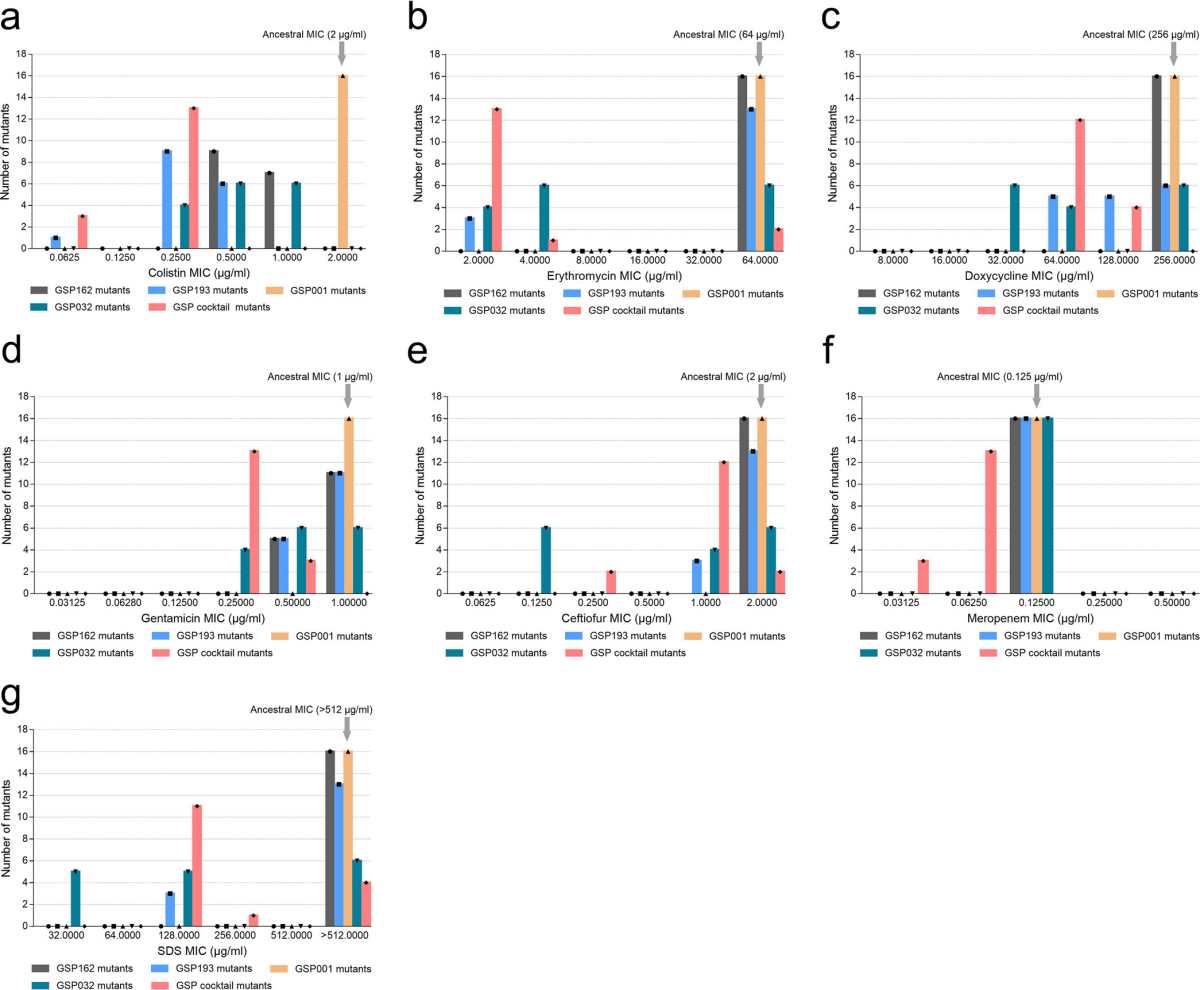
Antibiotic susceptibility of S. Enteritidis phage-resistant mutants. (a to g) MICs of colistin (a), erythromycin (b), doxycycline (c), gentamicin (d), ceftiofur (e), meropenem (f), and SDS (g) for S. Enteritidis mutants were determined using an adapted microdilution broth method in polypropylene 96-well plates at 37°C for 20 h. All 16 resistant mutants generated separately for each phage were assayed (detailed MIC data for all phage-resistant mutants are provided in Table S6). Selection for phage resistance results in a trade-off that significantly reduces the MIC. Three independent replicates were performed for each MIC.

Phage resistance mutations often involve the loss or modification of bacterial surface receptors. This may result in a fitness cost, such as reduced virulence, resensitization to antibiotics, and colonization defects (26). To gain insight into the costs associated with S. Enteritidis phage resistance, the growth kinetics and antibiotic resistance of all mutants were compared to those of the wild-type SE006 strain. First, we compared the MICs for resistant mutants with phage-sensitive parental strains of 6 clinically relevant antibiotics and SDS. As expected, phage-resistant mutants had various degrees of increased sensitivity to antibiotics and SDS compared to the parental strain (Fig. 6). All 16 phage-resistant mutants (phages GSP162, GSP193, and the phage cocktail) were more sensitive to colistin than the parental strain (Fig. 6a). Meanwhile, we also observed that mutants resistant to the phage cocktail were more sensitive to several antibiotics (colistin, erythromycin, doxycycline, gentamicin, ceftiofur, and meropenem) or SDS than the single-phage-resistant mutants. In contrast, the susceptibility of phage GSP001-resistant mutants to antibiotics or SDS had not altered. To determine whether the altered susceptibility of phage-resistant mutants to antibiotics is associated with changes in host bacterial surface receptors, the MICs for the S. Enteritidis SE006 mutants, in which the LPS synthesis genes and the OMP genes *btuB* and *tolC* were knocked out individually, were tested. We found that loss of certain LPS synthesis genes and the OMP gene *tolC* affects the susceptibility of bacteria to antibiotics (Fig. S6). These results are consistent with the susceptibility of phage-resistant mutants to antibiotics (Fig. 6). Our results suggest that the susceptibility of phage-resistant mutants to antibiotics is likely related to alterations of phage receptors.

**FIG 7:**
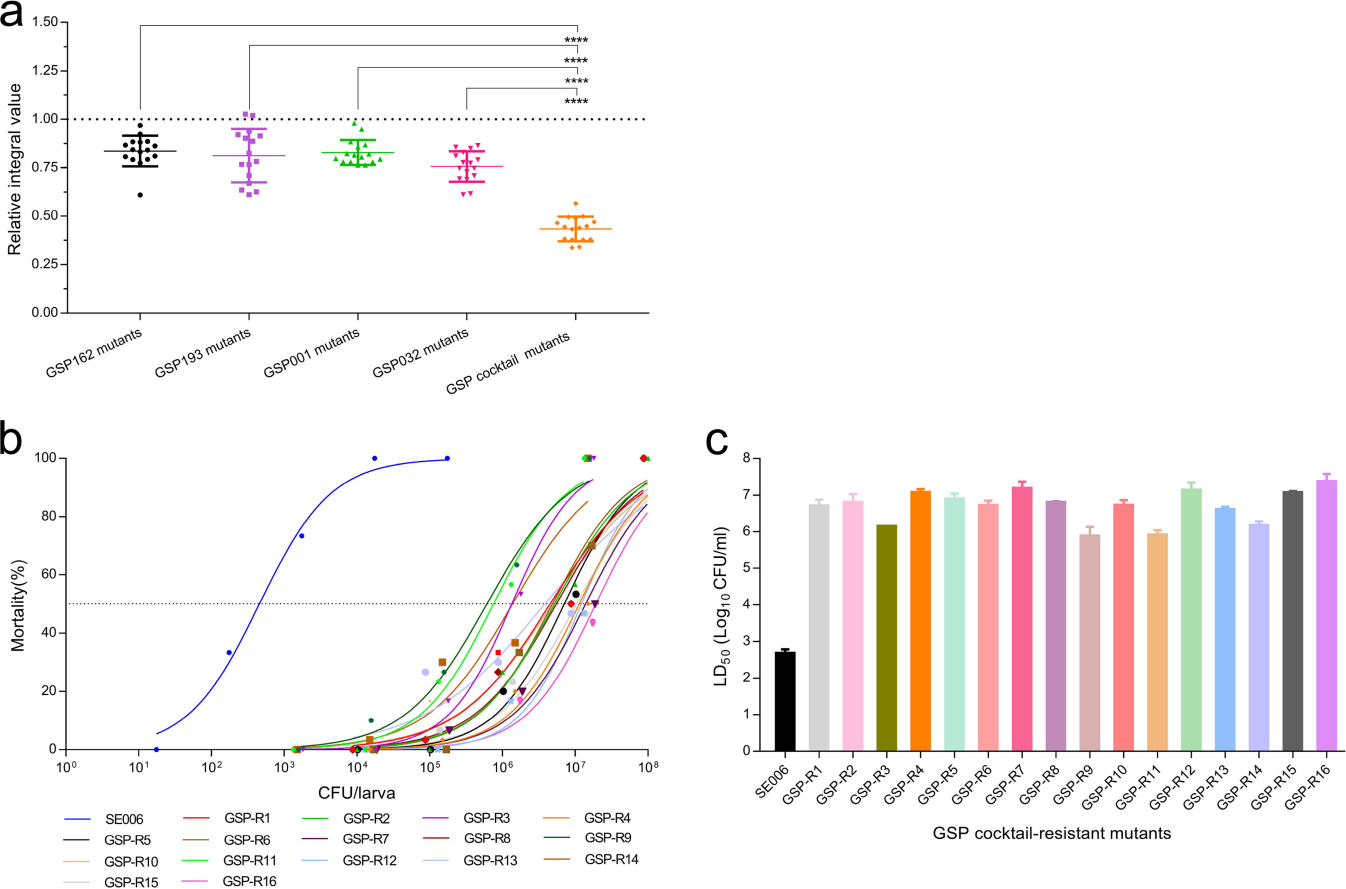
Phenotypic trade-offs of S. Enteritidis phage-resistant mutants. (a) Fitness costs associated with the growth of phage-resistant mutants. The relative integrals of growth curves of phage-resistant mutants represent estimates of relative fitness. The dashed line (relative fitness = 1) represents fitness equal to that of the parental SE006 strain. Growth curves and integrals of growth curves of each phage-resistant mutant are provided in Fig. S7. Statistical comparisons among different groups were performed using one-way ANOVA with Tukey’s multiple-comparison test (****, *P < *0.0001). (b and c) LD_50_ analysis of G. mellonella larvae infected with GSP (four-phage) cocktail-resistant mutants at 72 h. (b) Dose-response curves were plotted by using nonlinear regression analysis. (c) LD_50_s were determined based on the fitted curves. The LD_50_s of all the GSP cocktail-resistant mutants were significantly increased (at least 3 logs) compared to that of SE006. Three independent biological replicates were performed for each LD_50_.

We next assessed the growth ability of all resistant mutants *in vitro*. The total growth of bacterial strains was compared by assessing the integral of each growth curve (Fig. S7). We found that the relative integral of phage cocktail-resistant mutants was lower than that of the single-phage-resistant mutants (*P < *0.0001) (Fig. 7a). This observation suggested that phage-resistant mutants targeting different receptors simultaneously may result in more severe fitness costs, which reduce viability.

To further demonstrate reduced virulence of four phage cocktail-resistant mutants, we infected G. mellonella larvae with wild-type strains (SE006) or phage cocktail-resistant mutants (all 16 strains) and compared the 50% lethal doses (LD_50_s). As shown in Fig. 7b and c, the virulence of all phage cocktail-resistant mutants was significantly reduced, with an approximate 3-log increase in LD_50_ of phage cocktail-resistant mutants compared with the value for wild-type strains. As predicted, the fitness evolution of bacteria against phage cocktails results in significantly reduced virulence.

### Treatment with phage cocktails.

**FIG 8:**
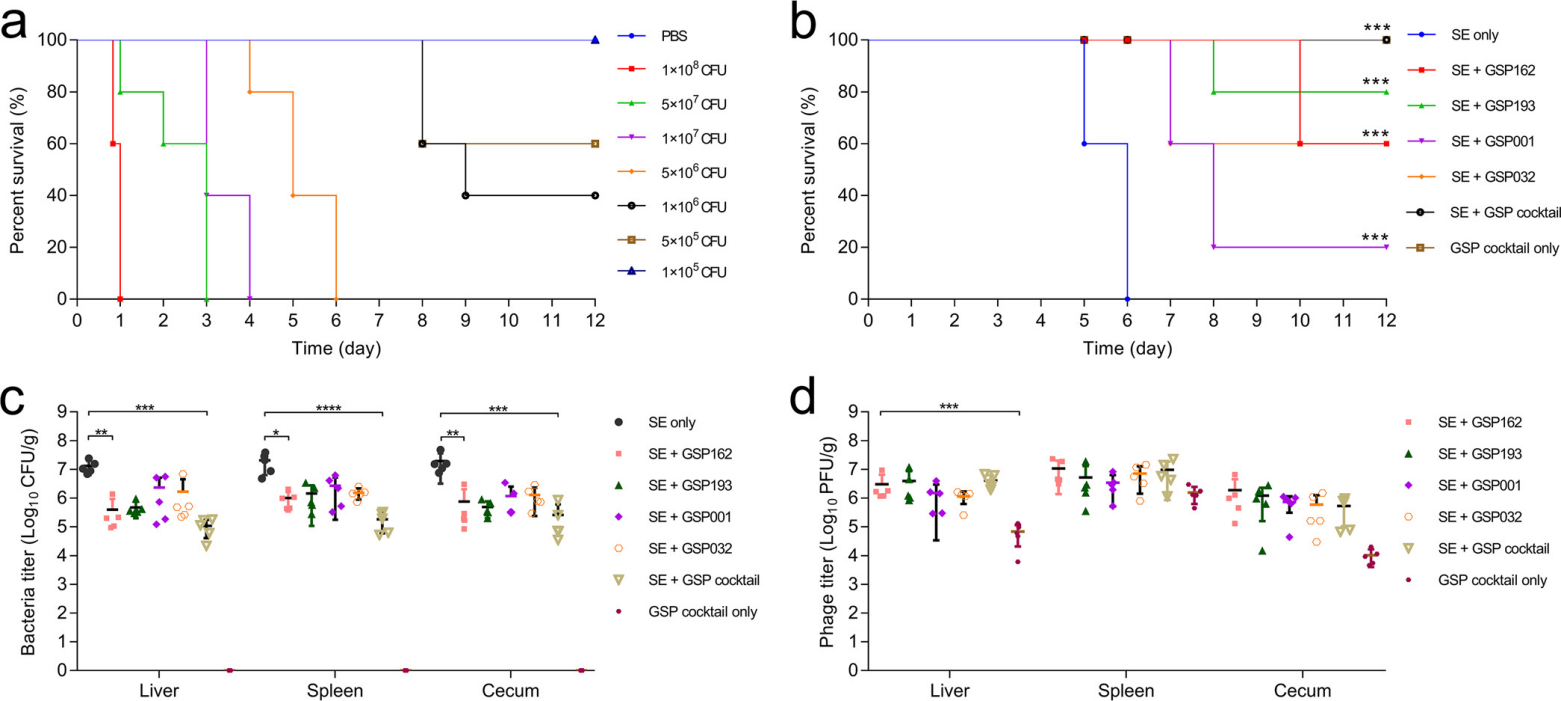
Protective effects of phage treatment against S. Enteritidis SE006 infection in mice. (a) Survival of mice with a single injection of SE006. The control group was injected with PBS. For each group, *n* = 5. (b) Survival of mice infected with SE006 (5 × 10^6^ CFU) followed by treatment with phage (MOI = 1) 1 h later. The control group was injected with PBS instead of bacteria or phage from the SE006-only and GSP cocktail-only groups, respectively. For each group, *n* = 5. ***, *P < *0.001 as determined using the log-rank (Mantel-Cox) test. (c and d) Bacterial CFU and phage PFU in mouse liver, spleen, and cecum at 24 h after phage treatment. For each group, *n* = 5. Each symbol represents data from one mouse. Bars denote group means. Statistical comparisons were performed, relative to the control (S. Enteritidis only or GSP cocktail only) using the Kruskal-Wallis test with Dunn’s *post hoc* multiple-comparison test (*, *P < *0.05; **, *P < *0.01; ***, *P < *0.001; ****, *P < *0.0001). SE, *S*. Enteritidis SE006; GSP cocktail, four-phage cocktail.

We next sought to evaluate the efficacy of phage therapy in controlling S. Enteritidis infection in mice. We first established the LD_100_ of S. Enteritidis strain SE006 (5 × 10^6^ CFU per mouse) in this mouse model (Fig. 8a). Mice were then infected with S. Enteritidis at the LD_100_ and subsequently treated with a single phage (GSP162, GSP193, GSP001, or GSP032) or a four-phage cocktail (GSP162+GSP193+GSP001+GSP032) via intraperitoneal (i.p.) injection 1 h after bacterial infection. Mice treated with single phages had significantly lower mortality rates than mice treated with phosphate-buffered saline (PBS), ranging from 20% to 80% survival. Importantly, treatment with the four-phage cocktail resulted in 100% survival of S. Enteritidis -infected mice (Fig. 8b). Furthermore, the titers of bacteria and phage in the liver, spleen, and cecum were determined for all groups of SE006-infected mice 24 h after phage treatment. Mice treated with phage showed a significant reduction in bacterial burden in the liver, spleen, and cecum compared to untreated mice (Fig. 8c). The median bacterial counts of four-phage cocktail groups (GSP cocktail) were lower than those for single-phage treatments in all tissues. Moreover, the phage titers in all bacterium-infected phage-treated groups were higher than those in mice that received phage only (Fig. 8d), suggesting that these phages may replicate within bacteria residing in mouse tissues. Taken together, our results indicated that phage treatment reduces mortality in SE006-infected mice and that this effect correlates with a reduction in bacterial load and the ability of phage to reproduce *in vivo*.

## DISCUSSION

Phage therapy has been recognized as a promising potential alternative to antibiotics in treating bacterial infections. The development of phage cocktails attempts to combat the evolution of phage resistance by combining multiple phages. However, phage cocktails should not be random mixtures of different phages but rather a rational selection of phages based on some evidence (27). In this study, we demonstrated that the bacterial receptor used by phages could be a criterion to aid in the design of cocktails. Four novel virulent phages targeting different receptors on S. Enteritidis (i.e., O antigen, LPS outer core, BtuB, and both TolC and LPS inner core) were identified by screening phages against a library of bacterial mutants (Fig. 1). We characterized them and determined that they have distinct host ranges (Fig. S2), phylogenies (Fig. S3c), and genome sequences (Fig. S8). These observations further suggested that the phages may employ different means to infect their hosts. Genomic analysis of these four phages did not identify any virulence or resistance genes. Altogether, these results indicated that the mixture of these four phages has synergistic therapeutic potential in treating bacterial infections.

We verified the synergistic effect of these four phages in killing S. Enteritidis by demonstrating that treatment with phage combinations targeting three or four different receptors resulted in significantly greater inhibition of bacterial growth than treatment with a single phage (Fig. 4). Notably, while combinations of different phages can inhibit the emergence of phage-resistant mutants to various degrees, we found that the combination of phages targeting four different receptors was the most stable in inhibiting host cell growth. These results are similar to those of previous studies showing that combinations of phages targeting different host receptors can reduce the likelihood and incidence of phage-resistant mutants (16, 28–30). For combinations of multiple phages, bacterial resistance to infection by a single phage may disrupt community structure, especially when individual resistance mutations provide cross-resistance to multiple phages (31). Therefore, we analyzed the evolution of cross-resistance in bacterium-phage interactions (Fig. 5). Our data show that single phage-resistant mutations cannot confer pleiotropic phage resistance against these four phages simultaneously, suggesting the synergistic effect of phages in lysing the host bacteria. In addition, resistant mutants generated against the same phage have different cross-resistance phenotypes, suggesting that independently evolved phage-resistant mutations based on host receptors may have different mutations conferring phage resistance. In response to phage infection, bacteria mainly rely on two types of defense mechanisms (32): constitutive (modification/masking of surface receptors) (11) and inducible (CRISPR-Cas immune system) (33). Constitutive defenses are more likely to evolve under ecological conditions with high phage density (32), and thus, surface receptor modifications may be highly ecologically dependent. This is consistent with bacteria producing phage-resistant mutants with different phenotypes.

In phage-host interactions, modification/masking of bacterial surface receptors to evolve constitutive defenses against phages is often associated with fitness costs (20, 34–37). To our knowledge, the type of fitness cost is mainly determined by the receptor type of the host, such as OMPs or LPSs. LPSs are a major surface component of Gram-negative bacteria and are one of the most potent microbial initiators of inflammation (38, 39). OMPs are also important virulence factors and play essential roles in bacterial infection (40). In some cases, those structures contribute to the prevention of environmental stress and to resistance to antibiotics (41, 42). In our study, we screened and identified phages GSP162, GSP193, GSP001, and GSP032 and found that the receptors necessary for infection were LPS (O antigen and inner and outer core) and outer membrane proteins (BtuB and TolC) (Fig. 2e). Although these phages had a clear synergistic effect on lysing of the host bacteria *in vitro*, mutants resistant to all four phages were still generated (Fig. 4). Therefore, we hypothesized that resistance to multiple phages is likely to be associated with greater fitness costs and that the fitness cost of multiple resistance mutations may be additive. Our results demonstrated that all mutants resistant to the four-phage cocktail exhibited reduced growth rates (Fig. S7), attenuated virulence (Fig. 7c), and increased sensitivity to antibiotics (Fig. 6). This indicated that the pressure from phages with different bacterial receptors drives the evolution of resistant bacterial mutants in a direction that entails more severe fitness costs. This finding is in line with previous research showing that an increased cost of phage resistance is linked to the evolutionary pressure exerted on the host bacteria (i.e., bacteria resistant to a single phage or multiple phages) and that bacteria accumulate multiple resistance mutations in different receptor genes to resist infection with all the phages present in the cocktail (43).

Our results suggest that phage-resistant mutants have regained susceptibility to antibiotics and SDS. Previous work has shown that drug susceptibility is related to the LPS barrier and the multidrug efflux system. LPS acts as a permeation barrier for Gram-negative bacteria to prevent the entry of hydrophobic antibiotics, detergents, and proteins (44, 45), while the bacterial multidrug efflux pump system can expel antibiotics, decontaminating agents, and dyes from the cell (46, 47). Our results also confirmed that inactivation of the efflux pump AcrAB-TolC or LPS truncation enhances drug sensitivity (Fig. S6), suggesting that the increased antibiotic susceptibility of phage-resistant mutants is closely linked to the phage host receptor. Moreover, the reduced virulence of phage-resistant mutants can also be explained by truncation of LPS, since LPS deletion results in severe virulence defects (48–50). Although we did not identify the site of specific genetic mutations in each phage-resistant strain, from an evolutionary perspective, we demonstrated that the evolutionary trade-off in phage-resistant mutants was influenced by phage receptor type.

Our analysis shows that the four-phage cocktail was more effective than each phage alone in killing S. Enteritidis *in vitro*. To test whether this effect also occurred *in vivo*, we evaluated the clinical effectiveness of phage therapy in a mouse model of systemic S. Enteritidis infection. Our results demonstrated that treatment with a four-phage cocktail increased the survival rate of SE006-infected mice compared to treatment with individual phages (Fig. 8b). Consistent with the mouse survival assay, the reduction of bacterial load in the liver, spleen, and cecal tissues also significantly decreased compared to that in the single-phage-treated groups (Fig. 8c), suggesting that the increased survival in mice was directly related to the phage-mediated killing of bacteria *in vivo*. However, it should be noted that CFU counts may be affected by the presence of phages in the tissue. Previous reports have demonstrated that phage therapy is an effective treatment in a mouse infection model (51–55). Our results suggest that phage cocktail therapy is more effective than single-phage therapy in the treatment of systemic S. Enteritidis SE006 infection in a mouse model.

In conclusion, we developed a novel phage cocktail consisting of four phages targeting different host receptors. which could delay the emergence of phage-resistant bacterial mutants. Our mouse model of S. Enteritidis infection also demonstrated that phage cocktail treatment was more effective than single-phage treatment in an animal model. Importantly, we found that phage cocktail-resistant mutants incurred more severe fitness costs than single phage-resistant mutants. These findings open the door for us to design intelligent phage cocktails and steer the evolution of phage-resistant strains toward clinically exploitable phenotypes.

## MATERIALS AND METHODS

### Bacterial strains and growth conditions.

Bacterial strains, plasmids, and phages used in this study are listed in Tables S1 and S2. All strains were cultured at 37°C in lysogeny broth (LB) or lysogeny broth agar (LA). All primers used in this study are listed in Tables S3 and S4.

### Phage library screen.

A total of 216 phages were isolated from various sources. Gene knockout S. Enteritidis SE006 strains used for phage receptor analysis to screen for phages targeting different receptors in the host are listed in Table S3. Briefly, an overnight culture of wild-type SE006 or knockout strains was mixed with 5 mL of LB soft agar (LB broth containing 0.7% [wt/vol] agar) and poured evenly onto LB agar plates. Phage solution was spotted onto the double-layer agar plates and incubated at 37°C overnight. After incubation, phages for which the formation of single plaques was observed in the library screen were then further validated by the efficiency of plating (EOP; phage titer of knockout strain/phage titer of wild-type SE006 strain).

### Assessment of phage lytic activity at different MOI.

The lytic activity of phages was analyzed in flat-bottomed 100-well microtiter plates by measuring optical density at 600 nm (OD_600_) every 30 min using the Bioscreen C system (Labsystems Oy, Helsinki, Finland). Briefly, 100 μL of S. Enteritidis SE006 (10^7^ CFU/mL) in exponential growth phase was mixed with either LB (control) or phage lysate over an MOI range of 10-fold dilutions between 10 and 0.000001 in a total volume of 100 μL. Bacterial growth was monitored at 37°C with shaking (200 rpm).

### *In vitro* phage cocktail killing curves.

To determine the lytic effect of different phage combinations on bacteria *in vitro*, the four selected phages were combined into communities in all possible combinations of single, double, triple, and quadruple phage mixtures using 100%, 50%, 33%, and 25% of each single-phage culture, respectively (Table S5) and then stored at 4°C.

The flat-bottomed 100-well microtiter plates were inoculated with S. Enteritidis SE006, and these cultures were incubated at 37°C with shaking (200 rpm) until early exponential phase (OD_600_ = 0.3 to 0.4). Cultures were then subsequently infected with each phage or all possible phage combinations at an MOI of 1 (approximately 10^8^ PFU/mL) and incubated at 37°C with shaking (200 rpm).

### Isolation of phage-resistant bacterial mutants.

Aliquots of 100 μL of overnight culture of wild-type SE006 strain (10^8^ CFU/mL) were incubated with 100 μL of phage (10^9^ PFU/mL) for 10 min at 37°C and subsequently plated on LB agar and incubated overnight at 37°C. At least 16 mutants resistant to phage GSP162, GSP193, GSP001, or GSP032 or the four-phage cocktail were obtained (80 in total) by selecting a single colony from each plate (Fig. S4). To ensure isolation of independent mutations, each mutant was sequentially streaked at least 3 times onto LB agar plates. All phage-resistant mutants were tested for resistance by spot testing on bacterial lawns and grown overnight in LB at 37°C with shaking. Because phage GSP001-resistant mutants were transiently resistant to reinfection with the phage, mutants were serially passaged using LB agar plates covered with GSP001 phage and grown in LB containing phage. All phage-resistant strains were stored frozen at −80°C in 20% glycerol.

### Bacteriophage host range determination.

To determine the host range of each phage, the EOP method was performed, as previously described (56). In brief, 10 μL of serial 10-fold dilutions of phage was spotted on the top of each bacterial lawn, and the plates were incubated overnight at 37°C. The EOP (phage titer of test bacteria/phage titer of host bacteria) was calculated.

### Cross-resistance assays.

To assess cross-resistance among phages GSP162, GSP193, GSP001, and GSP032, we cultured 16 mutants of each phage in parallel with and without the phage (control) at 37°C for 24 h using flat-bottomed 100-well microtiter plates. Growth of mutant strains was monitored by measuring the OD_600_ of the culture at 30-min intervals. The extent of cross-resistance is expressed as the RBG value (57), which was calculated by comparing the absorbance readings after 4 h of bacterial growth in the presence and absence of phage, according to the following formula: [*A*_600_(4 h) − *A*_600_(0 h)]*_bp_*/[*A*_600_(4 h) − *A*_600_(0 h)]*_b_*, where *b* denotes bacteria, *p* denotes the phage, and *A*_600_ is absorbance at 600 nm. RBG values are between 0 and 1, where values close to 1 represent full resistance (i.e., equal growth in the presence and absence of phage) and values close to 0 represent full susceptibility (i.e., lack of bacterial growth in the presence of phage). All assays were performed in triplicate.

### Antibiotic susceptibility assay.

MICs of antibiotics and sodium dodecyl sulfate (SDS) were determined using an adapted microdilution broth method (58). Briefly, an overnight culture of bacteria was diluted to ~10^6^ CFU/mL in fresh Mueller-Hinton broth (MHB) and was incubated in polypropylene 96-well plates with 2-fold serial dilutions of antibiotic for 20 h at 37°C. All tests were performed in triplicate, and the MIC was interpreted as the lowest concentration necessary to inhibit the growth of bacteria.

### Measuring the fitness costs associated with growth.

To determine fitness costs associated with the growth of phage-resistant bacterial mutants, the growth of all resistant mutants was compared to that of the parental SE006 strain. Ten microliters of overnight culture was inoculated into 190 μL of LB medium in flat-bottomed 100-well microtiter plates. Growth curves for each resistant mutant were determined by OD_600_ at 37°C with shaking (200 rpm). The integral of each growth curve was used to evaluate the growth ability of resistance mutants, according to Wright et al. (31). An estimate of relative fitness was calculated by dividing the integral of the growth curve of each resistant mutant by the average integral of the parental SE006 strain.

### Galleria mellonella larva infection model.

To compare the virulence of phage-resistant mutants and the parental SE006 strain, Galleria mellonella larvae were used as an infection model. The model has evaluated microbial pathogenicity and virulence for many bacterial pathogens (48, 59–61). Galleria mellonella larvae, 2.0 to 2.5 cm in length, were injected with serial dilutions of bacterial suspension (25 μL/larvae) in the left hindmost proleg using a 29-gauge insulin needle from Becton, Dickinson and Company (Franklin Lakes, NJ, USA). For each strain, 5 dilutions were made, and each dilution was injected into a group of 10 larvae for three biological replicates. Control larvae were injected with PBS alone. Larvae were then incubated at 37°C in the dark and were monitored for survival for up to 72 h. The median lethal dose (LD_50_) was calculated based on the regression curve analysis.

### Mouse infection model and ethics.

A mouse model of S. Enteritidis SE006 infection was developed using 6-week-old specific-pathogen-free (SPF) BALB/c female mice, which were purchased from the Experimental Animal Centre of Huazhong Agricultural University, Wuhan, China. The 100% minimum lethal dose (MLD_100_) of the SE006 strain was determined by inoculating groups of 5 mice intraperitoneally (i.p.) using 100 μL standard inoculum at various concentrations (1 × 10^5^, 5 × 10^5^, 1 × 10^6^, 5 × 10^6^, 1 × 10^7^, 5 × 10^7^, and 1 × 10^8^ CFU/mouse). One MLD_100_ was used in the mouse challenge assays. For phage therapy experiments, six groups of mice (10 per group) were challenged i.p. with 100 μL of SE006 (5 × 10^6^ CFU/mouse). After 1 h, mice in each group were injected i.p. with 100 μL of purified phage GSP162, GSP193, GSP001, GSP032, or GSP162+GSP193+GSP001+GSP032 (GSP cocktail) at an MOI of approximately 10. The bacterial control group (SE006 only) and phage control group (GSP cocktail only) were under the same conditions. The mortality of mice (5 per group) was regularly observed and recorded for 12 days.

To quantify phage and bacterial burden in tissues, mice were euthanized at 24 h after phage treatment. The liver, spleen, and cecum of each mouse (*n* = 5 per group) were then collected. Each tissue was homogenized in 1 mL of PBS. The titers of bacteria and phages in tissue homogenates were determined using plate count and double-layer plate methods, respectively. All protocols involving animals were performed with the approval of the Scientific Ethic Committee of Huazhong Agricultural University (no. HZAUMO-2022-0124). All mice were sacrificed by carbon dioxide asphyxiation at the end of the experiment.

### Data analysis.

Statistical analysis was carried out using GraphPad Prism 7.0 (GraphPad Software, San Diego, CA, USA). Experimental data are presented as means and standard deviations (SD) from three independent experiments. The survival of infected mice was analyzed using the log-rank (Mantel-Cox) test. All other comparisons were performed using one-way analysis of variance (ANOVA) with Tukey’s multiple-comparison test or the nonparametric Kruskal-Wallis test with Dunn’s multiple-comparison test. LD_50_ values were calculated using nonlinear regression analysis.

### Data availability.

The complete genome sequences of bacteria and phages have been deposited in GenBank and assigned the following accession numbers: CP099973 (S. Enteritidis SE006), ON855038 (phage GSP193), ON855039 (phage GSP001), ON855040 (phage GSP032), and ON855041 (phage GSP162).
