# Supplementary material for: Fitness Trade-Offs in Phage Cocktail-Resistant Salmonella enterica Serovar Enteritidis Results in Increased Antibiotic Susceptibility and Reduced Virulence
Source: Microbiol Spectr. 2022 Sep 27;10(5):e02914-22. doi: 10.1128/spectrum.02914-22 (PMC9603643; doi:10.1128/spectrum.02914-22)
Supplement: Supplemental file 1 — Supplemental material. Download spectrum.02914-22-s0001.pdf, PDF file, 6.7 MB [file spectrum.02914-22-s0001.pdf]

Fitness trade-offs in phage cocktail-resistant *Salmonella* Enteritidis results in increased antibiotic susceptibility and reduced virulence

Dongyang Gao, Hongyue Ji, Linkang Wang, Xinxin Li, Dayue Hu, Junna Zhao, Shuang Wang, Pan Tao, Xiangmin Li, Ping Qian

Supplementary Information

Supplementary Figures 1-8.

Supplementary Tables 1-6.

RESULTS

Morphological and genomic characteristics of four selected phages. The plaque morphology of phages observed on the bacterial lawn showed that all four phages were able to form clean and bright plaques and had a high infection efficiency (Supplementary Fig. 2a). Transmission election microscopy (TEM) revealed that all four phages belong to the order *Caudovirales* (Supplementary Fig. 2b). Phages GSP162, GSP001, and GSP032 had a polyhedral head and a non-contractile tail, and phage GSP193 had a polyhedral head and a contractile tail. The phylogenetic analysis using protein sequences of terminase large subunit indicated that phages GSP162, GSP193, GSP001, and GSP032 belong to the *Siphoviridae*, *Myoviridae*, *Demerecviridae*, and *Drexlerviridae* family, respectively (Supplementary Fig. 2c). Sequencing results showed that all the phages had a double-stranded DNA genome ranging from 43 kb to 114 kb (Supplementary Fig. 8a, b, c, d). No genes related to lysogeny were found in the genomes (1), indicating that all these phages are lytic and might be candidates for phage therapy. Moreover, no genes associated with antimicrobial resistance and virulence were detected in the phage genomes using the ResFinder and VirulenceFinder databases.

MATERIALS AND METHODS

Gene knockout and complementation. All *S.* Enteritidis SE006 derivatives

with deletions of specific genes were generated using the lambda Red recombinase system, as previously described (2). Electrocompetent *S.* Enteritidis SE06 cells were then transformed with the pkD46-Cm^r (pkD46 containing chloramphenicol resistance gene) plasmid via electroporation. Afterward, the kanamycin resistance cassette was obtained from pkD4 using primers specified in Supplementary Table 3. Subsequently, the PCR product was electroporated into the SE006-pKD46-Cm^r electrocompetent cells, and then specific genes were replaced with kanamycin cassettes. Finally, all mutants with specific gene deletions were verified by PCR amplification and subsequent DNA sequencing.

To complement specific genes back into knockout mutants, target genes were amplified using primers (Supplementary Table 4) containing a recognition site of the restriction endonuclease. Next, the PCR fragments were cloned into the plasmid pHSG396, and this recombinant plasmid was electroporated into corresponding knockout mutants. All complementary strains were confirmed by PCR.

Phage isolation and purification, and transmission electron microscopy. Using the *S.* Enteritidis SE006 strain as host bacteria, phages were isolated from various sources through the double-layer agar method as previously described (3). A highly concentrated phage lysate was purified by CsCl gradient ultracentrifugation (4). Purified phage particles were dropped on carbon film-coated copper grids (Carbon Type-B 200 mesh; Beijing

Zhongjingkeyi Technology Co., Ltd., Beijing, China) and visualized by transmission electron microscopy (TEM) (H-7650, Hitachi, Tokyo, Japan) with an accelerating voltage of 100 kV. The phage lysates were stored at 4°C.

Genome analysis and phylogenetic analysis. Whole-genome sequencing of *S*. Enteritidis SE006 strain and phages GSP162, GSP193, GSP001, and GSP032 was performed using the Illumina MiSeq (San Diego, CA, USA) system. The phage genome sequences were annotated using RAST (http://rast.nmpdr.org/) and were manually checked using by BLASTp (https://blast.ncbi.nlm.nih.gov/Blast.cgi). Potential tRNA genes in the phage genome sequence were identified by using tRNAscan-SE (http://lowelab.ucsc.edu/tRNAscan-SE/) (5). Phage genomes were analyzed for potential antibiotic resistance and virulence genes by ResFinder (https://cge.cbs.dtu.dk/services/ResFinder/) and VirulenceFinder (https://cge.cbs.dtu.dk/services/VirulenceFinder/) (6), respectively. Lysogeny-associated proteins were manually validated via annotated phage genomes. The circular map of the phage whole-genome was constructed using CGView (http://stohard.afns.ualberta.ca/cgview_server/) (7). To determine the taxonomy of the four phages, sequences belonging to the terminase large subunits of different phages according to the International Committee on Taxonomy of Viruses (ICTV) classification (https://talk.ictvonline.org/taxonomy/) reports were downloaded from the NCBI database. Phylogenetic trees based on the terminase large subunits of four

phages were constructed in MEGA version 7.0 using the neighbor-joining method (8).

REFERENCES

1. Oppenheim AB, Kobiler O, Stavans J, Court DL, Adhya S. 2005. Switches in bacteriophage lambda development. Annu Rev Genet 39:409-29.
2. Datsenko KA, Wanner BL. 2000. One-step inactivation of chromosomal genes in Escherichia coli K-12 using PCR products. Proc Natl Acad Sci U S A 97:6640-5.
3. Chen Y, Sun E, Song J, Tong Y, Wu B. 2018. Three Salmonella enterica serovar Enteritidis bacteriophages from the Siphoviridae family are promising candidates for phage therapy. Can J Microbiol 64:865-875.
4. Tao P, Li Q, Shivachandra SB, Rao VB. 2017. Bacteriophage T4 as a Nanoparticle Platform to Display and Deliver Pathogen Antigens: Construction of an Effective Anthrax Vaccine. Methods Mol Biol 1581:255-267.
5. Schattner P, Brooks AN, Lowe TM. 2005. The tRNAscan-SE, snoscan and snoGPS web servers for the detection of tRNAs and snoRNAs. Nucleic Acids Res 33:W686-9.
6. Kleinheinz KA, Joensen KG, Larsen MV. 2014. Applying the ResFinder and VirulenceFinder web-services for easy identification of acquired antibiotic resistance and E. coli virulence genes in bacteriophage and prophage nucleotide sequences. Bacteriophage 4:e27943.
7. Grant JR, Stothard P. 2008. The CGView Server: a comparative genomics tool for circular genomes. Nucleic Acids Res 36:W181-4.
8. Kumar S, Stecher G, Tamura K. 2016. MEGA7: Molecular Evolutionary Genetics Analysis Version 7.0 for Bigger Datasets. Mol Biol Evol 33:1870-4.

Supplementary Figures and legends

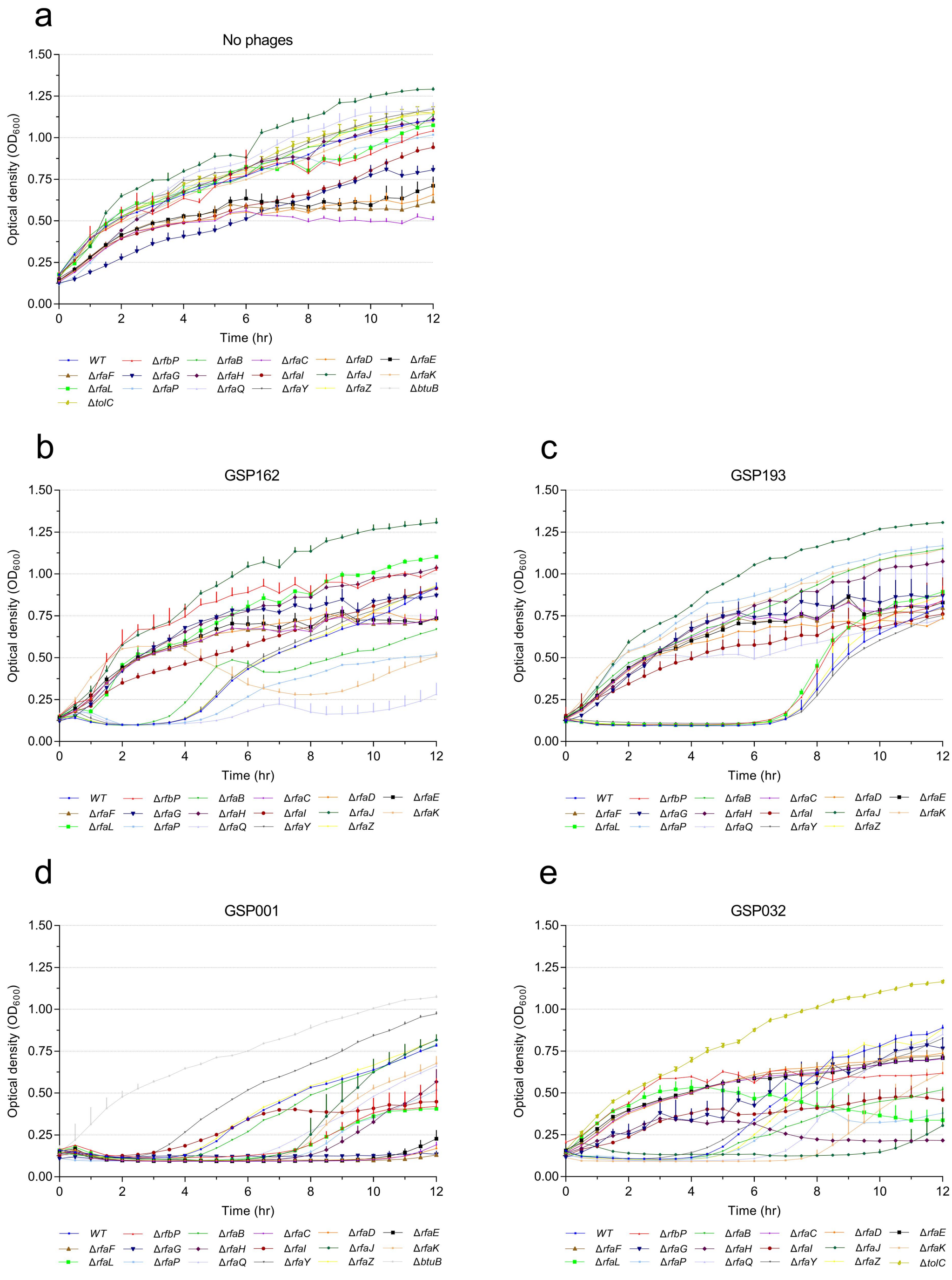

Supplementary Fig. 1 The ability of phage to lyse phage receptor-related

gene knockout strains in a liquid medium. a Growth curves of wild-type SE006 and gene knockout strains in the absence of phage. **b** Bacterial growth curve shows that phage GSP162 can completely lyse wild-type strains and the gene knockout strains of *rfaB, rfaP, rfaQ, rfaY,* and *rfaZ*. **c** Bacterial growth curve shows that phage GSP193 can completely lyse wild-type strains and the gene knockout strains *of rfbP, rfaL, rfaY,* and *rfaZ*. **d** Bacterial growth curves show that phage GSP001 can completely lyse wild-type strains and all LPS synthesis-related gene knockout strains, but not *btuB* gene knockout strains. **e** Bacterial growth curve shows that phage GSP032 can completely lyse wild-type strains and the gene knockout strains of *rfaB, rfaJ, rfaI, rfaK, rfaP, rfaQ, rfaY*, and *rfaZ*.

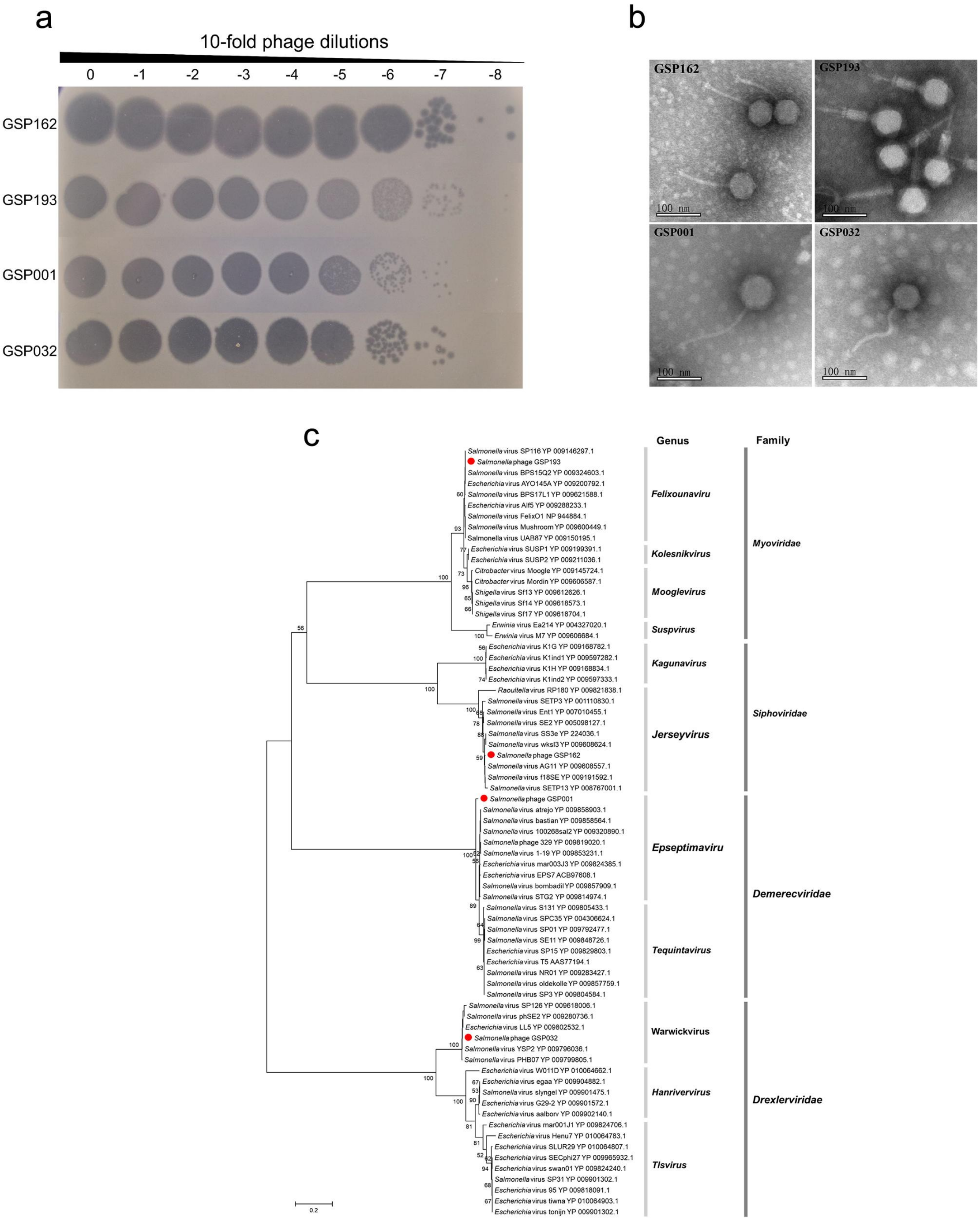

Supplementary Fig. 2 Morphology and genomic characteristics of phages GSP162, GSP193, GSP001, and GSP032. a 10-fold serial dilutions of phage GSP162, GSP193, GSP001, and GSP032 were spotted on lawns of *S.* Enteritidis SE006 strain. Clear plaques were observed. **b** TEM of phages GSP162, GSP193, GSP001 and GSP032. Scale bar, 100 nm. **c** Comparative

analysis of phages GSP162, GSP193, GSP001, and GSP032 using terminase large subunit sequences. Amino acid sequences were aligned using MEGA 7.0 software, and the phylogenetic tree was constructed using maximum likelihood with 1000 bootstrap replications.

Bacteria and serovars	Stain no.	GSP 162	GSP 193	GSP 001	GSP 032
S. Enteritidis	SE001	+++	+++	+++	-
	SE002	+++	+++	+++	+++
	SE003	+++	+++	+++	+++
	SE004	+++	+++	+++	+++
	SE005	+++	+++	+++	+++
	SE006	+++	+++	+++	+++
	SE007	+++	+++	+++	+++
	SE008	+++	+++	+++	+++
	GS001	+++	+++	+++	+
	GS028	+++	+++	+++	+
	ATCC 17036	+++	+++	+++	+
S. Typhimurium	ST001	+++	+++	+	-
	ST002	++	+++	+++	-
	ST003	++	+++	+++	-
	ST004	+	+++	+++	-
	ST005	-	+++	++	-
	ST006	+	++	+	+
	ST007	+	+++	+++	-
	ST008	+	++	+	+
	GS007	+	+++	+++	-
	GS026	+	+++	+	+
	ATCC 14028	+	+++	++	+
	SL1344	+	+++	++	+
S. Pullorum	SP001	-	+++	+++	+++
	SP002	+++	+++	+++	+
	SP003	+	+++	+++	+++
	SP004	+++	+++	+++	+++
	SP005	+++	+++	+++	+++
	SP006	+	+++	+++	+++
	SP007	+++	+++	+++	+++
	SP008	+	+++	+++	+++
	GS025	+++	+++	+	+++

Bacteria and serovars	Stain no.	GSP 162	GSP 193	GSP 001	GSP 032
S. Corvallis	GS002	-	+	-	-
S. Derby	GS003	+	+++	-	-
S. Rissen	GS004	-	+	-	-
S. Weltevreden	GS006	-	+++	+++	-
S. Panama	GS008	+	+	+	+
S. Cerro	GS009	-	+++	++	-
S. Havana	GS010	-	+	-	+
S. Kentucky	GS011	-	+	-	+
S. Goldcoast	GS013	-	++	-	-
S. Meleagridis	GS014	-	+++	-	-
S. Agona	GS015	+	+++	++	++
S. India	GS016	+	+++	++	-
S. Saintpaul	GS017	+	++	++	-
S. Yphi	GS027	-	+++	+++	-
S. Para-typhi	GS029	-	+++	++	+
S. Choleraesuis	ATCC 10708	+	++	++	+
Escherichia coli	BL21	-	-	-	++
	DH5α	-	-	+	++
	ATCC 35150	-	-	-	-
	ATCC 25922	-	-	-	-
Listeria monocytogenes	ATCC 19111	-	-	-	-
Staphylococcus aureus	ATCC 25923	-	-	-	-
Klebsiella pneumoniae	ATCC 70603	-	-	-	-

+++	0.5 ≤ EOP ≤ 1.5
++	0.01 < EOP < 0.5
+	0.0001 ≤ EOP ≤ 0.01
-	Not susceptible to phages

Supplementary Fig. 3 Host range analysis of phages GSP162, GSP193, GSP001, and GSP032. Host range was determined by EOP. +++, 0.5 ≤ EOP ≤ 1.5; ++, 0.01 < EOP < 0.5; +, 0.0001 ≤ EOP ≤ 0.01; -, Not susceptible to phages. ATCC, American Type Culture Collection.

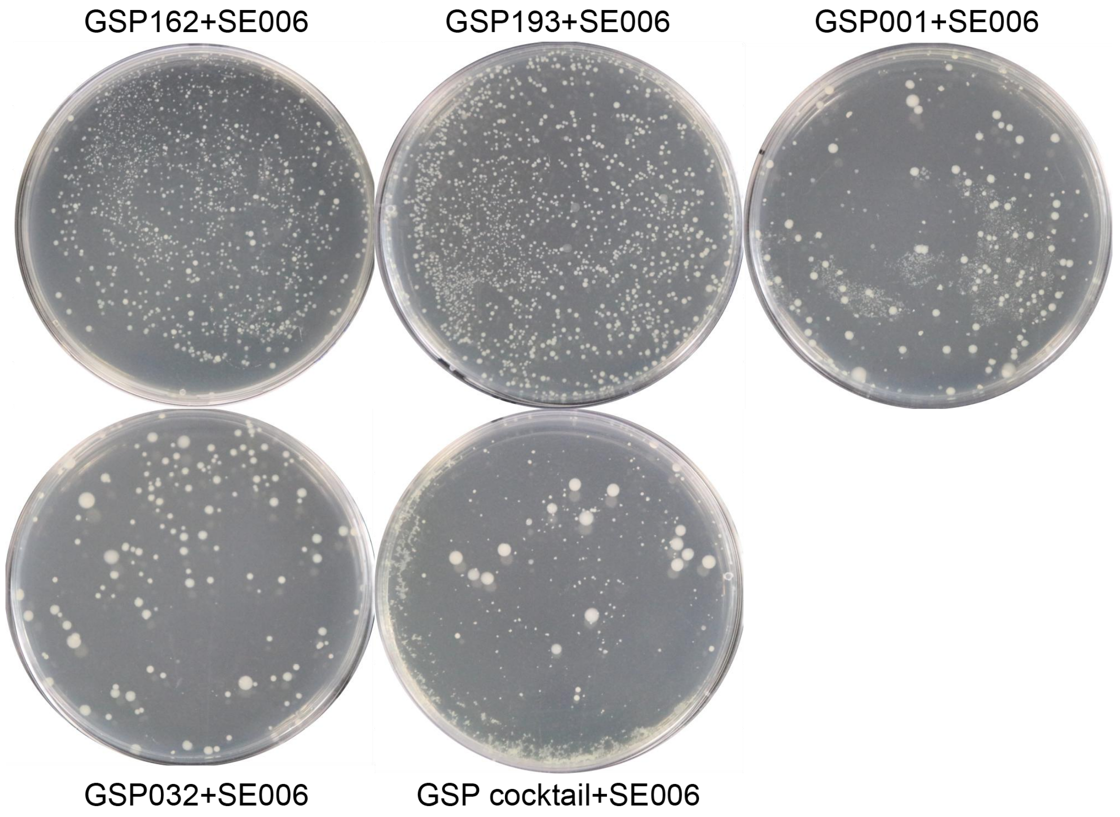

Supplementary Fig. 4 Spontaneous phage-resistant mutants derived from *S.* Enteritidis SE006 strain have distinct morphologies. *S.* Enteritidis SE006 strain was incubated with phage GSP162, GSP193, GSP001, GSP032, or GSP cocktail for 10 min, and then inoculated on LB agar plates at 37°C for 48h. GSP cocktail, four-phage cocktail.

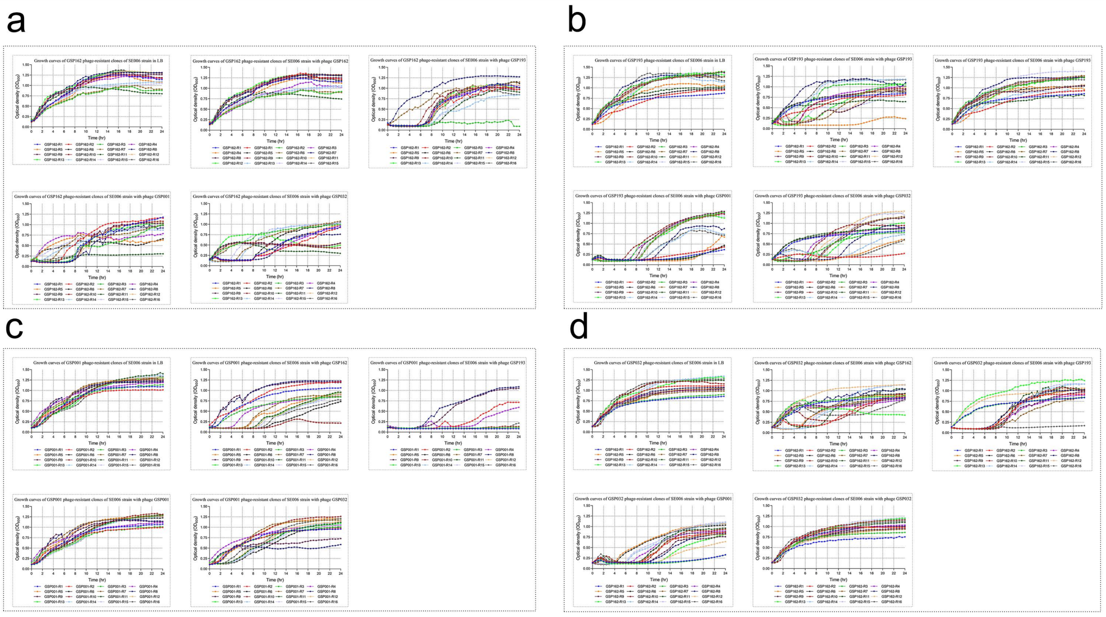

Supplementary Fig. 5 Cross-resistance analysis. a to **d** Growth curves of phage-resistant mutants treated with phage GSP62 (a), GS193 (b), GS0001 (c), and GSP032 (d), respectively. Bacteria were grown for 24 hours in the presence or absence of each phage (at MOI of 10). The initial inoculum concentration of bacteria was approximately 10^7 CFU/mL. Optical density (OD at 600 nm) of bacteria was measured every 30 min. Data are representative of three independent experiments and represented as mean values.

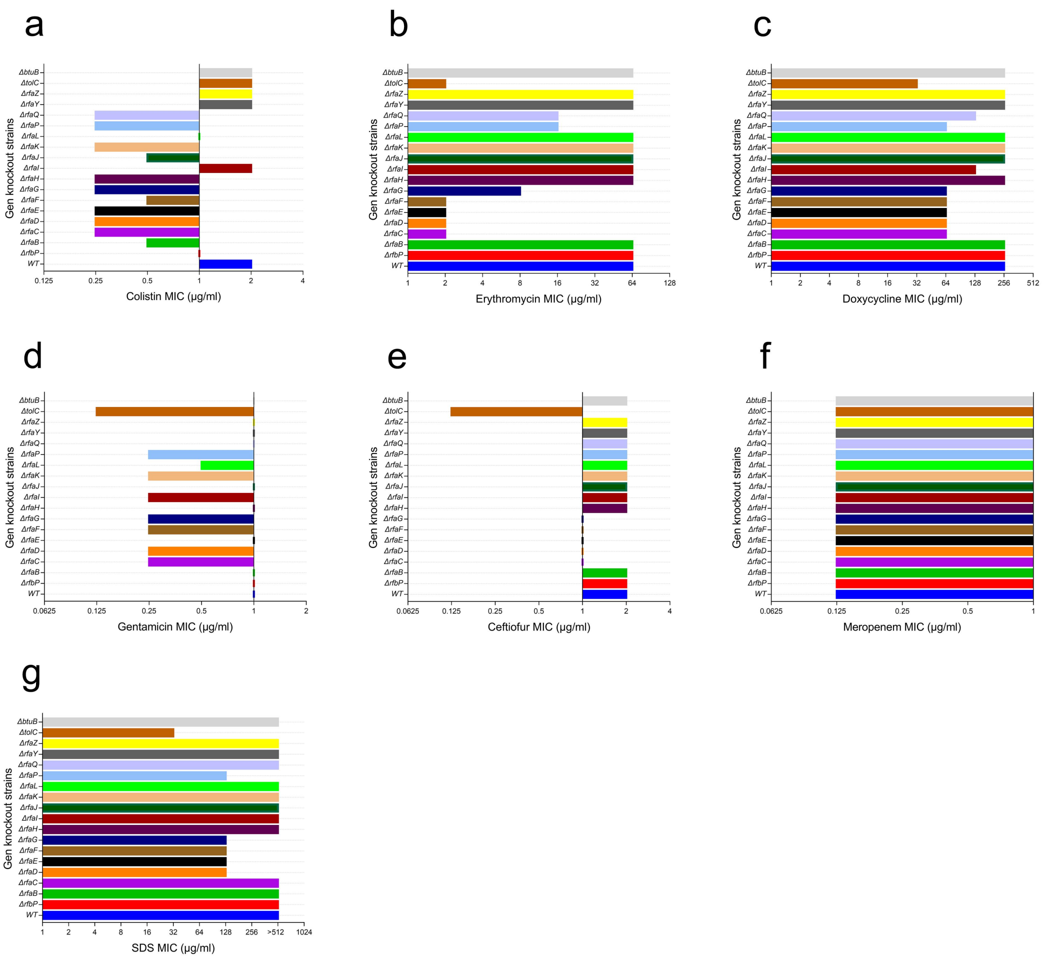

Supplementary Fig. 6 The MIC (μg/mL) of single knockout strains associated with phage receptors. The MIC of colistin (**a**), erythromycin (**b**), doxycycline (**c**), gentamicin (**d**), ceftiofur (**e**), meropenem (**f**), and SDS (**g**) towards knockout strains were determined using an adapted microdilution broth method in polypropylene 96-well plates at 37 for 20h. Three independent replicates were performed for each MIC value.

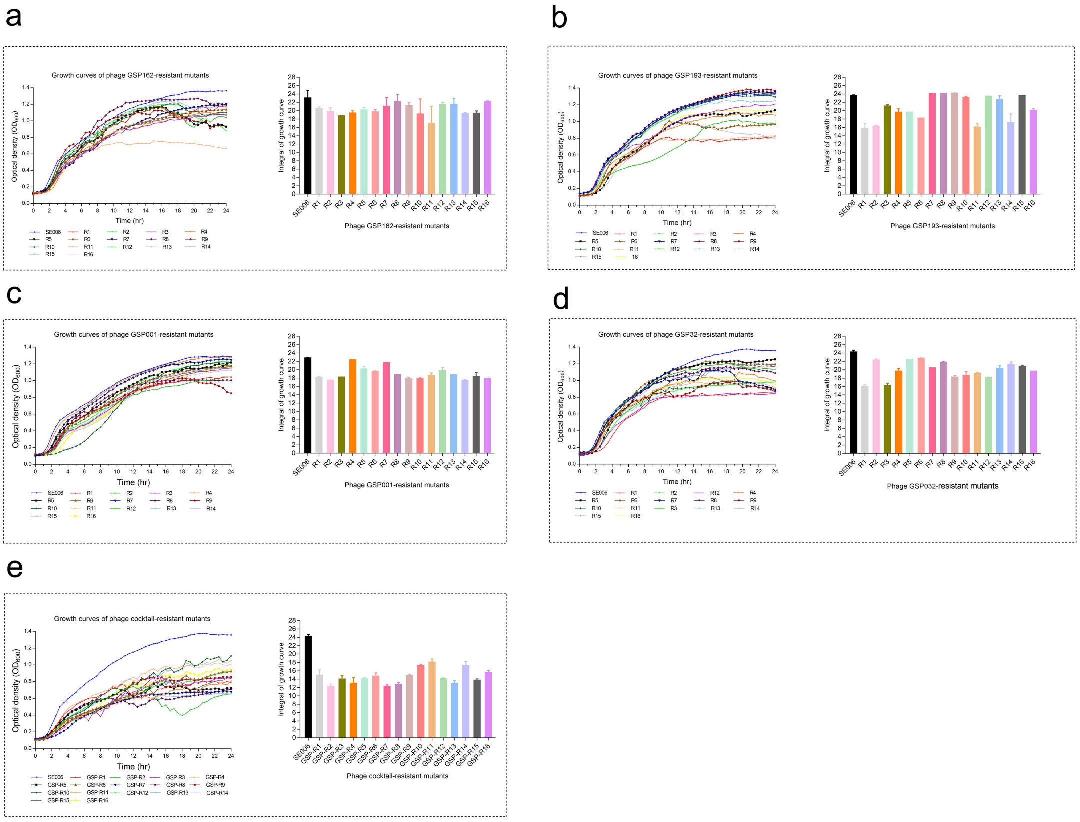

Supplementary Fig. 7 Growth of phage-resistant mutants in liquid medium. a to **e** The integral of the growth curve as an area under the curve was used to compare the growth ability of GSP162 (**a**), GSP193(**b**), GSP001(**c**), and GSP032(**d**) phage-resistant mutants.

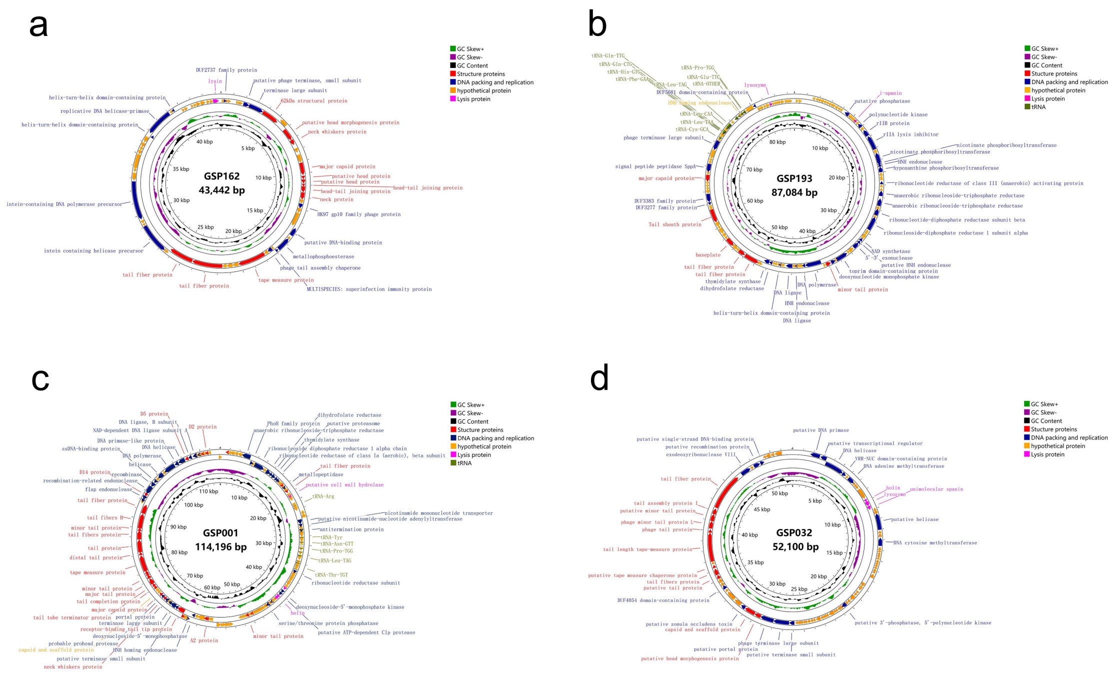

Supplementary Fig. 8 Genome analysis of phages. a to **d** Circular genome map of phage GSP162 (a)， GSP193 (b), GSP001 (c), and GSP032 (d). The functions of the open reading frame (ORF) are represented with specific colors according to their functional categories. The map was generated using the CGview server.

Supplementary Tables

Supplementary Table 1 Bacterial strains used in this study.

Bacterial Strains	Strain ID Number	Source of Strains
Salmonella enterica serovar Enteritidis (*S.* Enteritidis)	SE001	Lab Stock
	SE002	Lab Stock
	SE003	Lab Stock
	SE004	Lab Stock
	SE005	Lab Stock
	SE006	Lab Stock
	SE007	Lab Stock
	SE008	Lab Stock
	GS001	Lab Stock
	GS028	Lab Stock
	ATCC17036	ATCC
Salmonella enterica serovar Typhimurium (*S.* Typhimurium)	ST001	Lab Stock
	ST002	Lab Stock
	ST003	Lab Stock
	ST004	Lab Stock
	ST005	Lab Stock
	ST006	Lab Stock
	ST007	Lab Stock
	ST008	Lab Stock
	GS007	Lab Stock
	GS026	Lab Stock
	ATCC14028	ATCC
	SL1344	Lab Stock
Salmonella enterica serovar Pullorum (*S.* Pullorum)	SP001	Lab Stock
	SP002	Lab Stock
	SP003	Lab Stock
	SP004	Lab Stock
	SP005	Lab Stock
	SP006	Lab Stock
	SP007	Lab Stock
	SP008	Lab Stock
	GS025	Lab Stock
Salmonella enterica serovar Corvallis (*S.* Corvallis)	GS002	Lab Stock
Salmonella enterica serovar Derby (*S.* Derby)	GS003	Lab Stock
Salmonella enterica serovar Rissen (*S.* Rissen)	GS004	Lab Stock
Salmonella enterica serovar Weltevreden (*S.* Weltevreden)	GS006	Lab Stock

Salmonella enterica serovar Panama (*S.* Panama)	GS008	Lab Stock
Salmonella enterica serovar Cerro (*S.* Cerro)	GS009	Lab Stock
Salmonella enterica serovar Havana (*S.* Havana)	GS010	Lab Stock
Salmonella enterica serovar Kentucky (*S.* Kentucky)	GS011	Lab Stock
Salmonella enterica serovar Goldcoast (*S.* Goldcoast)	GS013	Lab Stock
Salmonella enterica serovar Meleagridis (*S.* Meleagridis)	GS014	Lab Stock
Salmonella enterica serovar Agona (*S.* Agona)	GS015	Lab Stock
Salmonella enterica serovar India (*S.* India)	GS016	Lab Stock
Salmonella enterica serovar Saintpaul (*S.* Saintpaul)	GS017	Lab Stock
Salmonella enterica serovar Yphi (*S.* Yphi)	GS027	Lab Stock
Salmonella enterica serovar Para-typhi (*S.* Para-typhi)	GS029	Lab Stock
Salmonella enterica serovar Choleraesuis (*S.* Choleraesuis)	ATCC 10708	ATCC
Escherichia coli	BL21	Lab Stock
	DH5α	Lab Stock
	ATCC 35150	ATCC
	ATCC 25922	ATCC
Listeria monocytogenes	ATCC 19111	ATCC
Staphylococcus aureus	ATCC 25923	ATCC
Klebsiella pneumoniae	ATCC 7060	ATCC

ATCC, American Type Culture Collection

Supplementary Table 2 Plasmids used in this study.

Plasmid	Description	References or Source
pKD46, Cm^r	Red recombinase expression plasmid was modified to Cm^r	Lab stock
pKD4	Template plasmid containing FRT-flanked cat, kan^r	Lab stock
pHSG396	Low copy clone vector, Cm^r	Lab stock

Supplementary Table 3 Knockout strain primers used in this study.

Primer	Nucleotide sequence [5`→ 3`]	Gene knockout strain	References
SE006-*rfbP*-red-F	ATGGATAATATTGATAATAAGTATAATCCACAGCTATGTTGTAGGCTGGAGCTGCTTC	SE006, Δ*rfbP*::kan^r	This study
SE006-*rfbP*-red-R	TTAATACGCACCATCTCGCCGCAAAACAACTTTCGCTGTATGGGAATTAGCCATGGTCC		
SE006-*rfaB*-red-F	CTAAAGTCTTTAGATTAAGGTTTTAACTCTGGGGTATATTGTAGGCTGGAGCTGCTTC	SE006, Δ*rfaB*::kan^r	This study

SE006-*rfaB*-red-R	TCACTTTTGTAATTTCGAGAATATTGCATTATTAAAATTATGGGAATTAGCCATGGTCC		
SE006-*rfaC*-red-F	CGGGTTTTGATCGTTAAAACATCATCGATGGGCGACGTATGTAGGCTGGAGCTGCTTC	SE006, Δ*rfaC*::kan^r	This study
SE006-*rfaC*-red-R	CTTTTCTCCACAATAGGTTTGGGATGAGACAGAGTCTCTATGGGAATTAGCCATGGTCC		
SE006-*rfaD*-red-F	CGCCCACCCAGGACGGGCCAATGACCAAAATTTTCATACTGTAGGCTGGAGCTGCTTC	SE006, Δ*rfaD*::kan^r	This study
SE006-*rfaD*-red-R	TGAGACAGTCTCTGACACCATAATTCAAAGGTTACAGTTATGGGAATTAGCCATGGTCC		
SE006-*rfaE*-red-F	ATGAAAGTAAATCTGCCAGCGTTTGAACGTGCAGGCGTCTGTAGGCTGGAGCTGCTTC	SE006, Δ*rfaE*::kan^r	This study
SE006-*rfaE*-red-R	TTACTTCTCGCTCTCGGTCTGGATCTTTTTGATGATATTATGGGAATTAGCCATGGTCC		
SE006-*rfaF*-red-F	TTAAACGCCCTCTTCCGACAACAGCGAATGAAGCTCTTCTGTAGGCTGGAGCTGCTTC	SE006, Δ*rfaF*::kan^r	This study
SE006-*rfaF*-red-R	ATGAAAATTTTGGTCATTGGCCCGTCCTGGGTGGGCGACATGGGAATTAGCCATGGTCC		
SE006-*rfaG*-red-F	GCGCCATAGTGTGGTTAACGGCGCTTTCAGCTCAACCATTGTAGGCTGGAGCTGCTTC	SE006, Δ*rfaG*::kan^r	This study
SE006-*rfaG*-red-R	CATGAGGCACGCACCATAGATTTGGACAGCCTGCTATGAATGGGAATTAGCCATGGTCC		
SE006-*rfaH*-red-F	CCGAACAAGCGGATAAGAGTCATTATGCAATCCTGGTATTGTAGGCTGGAGCTGCTTC	SE006, Δ*rfaH*::kan^r	This study
SE006-*rfaH*-red-R	AACGCCAGAACCTTATTTGAGGTCGTATTCTGAACGATAGATGGGAATTAGCCATGGTCC		
SE006-*rfaI*-red-F	ATGAGCAGAAAATATTTTGAAGAAGAAGTCATTCAACAGTGTAGGCTGGAGCTGCTTC	SE006, Δ*rfaI*::kan^r	This study
SE006-*rfaI*-red-R	TTATTCAAGAAGTTTACGTTTAAAGTAGGCTATATAGTAATGGGAATTAGCCATGG		

	TCC		
SE006-*rfaJ*-red-F	GTAACTTATTTTTGCCAAAATTTTGGATACAGAATAAATTGTAGGCTGGAGCTGCTTC	SE006, Δ*rfaJ*::kan^r	This study
SE006-*rfaJ*-red-R	TTATTATATATCATAATGAATTATTTTAACCTTAAATCTATGGGAATTAGCCATGGTCC		
SE006-*rfaK*-red-F	CCTCCACGTGGCGCGGCTGCGGTAGAAACCTGGATTTACTGTAGGCTGGAGCTGCTTC	SE006, Δ*rfaK*::kan^r	This study
SE006-*rfaK*-red-R	TTACGGTATCAGCGCGTTTTCCATCGTCCGGACTCAATCATGGGAATTAGCCATGGTCC		
SE006-*rfaL*-red-F	AGAGACTCTGTCTCATCCCAAACCTATTGTGGAGAAAAGTGTAGGCTGGAGCTGCTTC	SE006, Δ*rfaL*:kan^r	This study
SE006-*rfaL*-red-R	AAAACGCGCTGATACCGTAATAAGTATCAGCGCGTTTTTATGGGAATTAGCCATGGTCC		
SE006-*rfaP*-red-F	AAGGCCGCGGATATTATTACAGGTGATTTAGATGGTTGAGTGTAGGCTGGAGCTGCTTC	SE006, Δ*rfaP*::kan^r	This study
SE006-*rfaP*-red-R	CTTTCTTTAATTCGAGCGGCTTTAACATCCGCCTGGTGAAATGGGAATTAGCCATGGTCC		
SE006-*rfaQ*-red-F	CTTGTGGAAAAGCCATTTCGAAGAATTCTGATTATAAAATGTAGGCTGGAGCTGCTTC	SE006, Δ*rfaQ*::kan^r	This study
SE006-*rfaQ*-red-R	AGCAGGCTGTCCAAATCTATGGGGCGTGCCTCATGCGGCATGGGAATTAGCCATGGTCC		
SE006-*rfaY*-red-F	TGCGTTATAGTGTTTTCATCATAAGAGCAGGAGAACACATGTAGGCTGGAGCTGCTTC	SE006, Δ*rfaY*::kan^r	This study
SE006-*rfaY*-red-R	GCTCACGATGTCGTTCCCATCGCAGATAAGAGAAAGGGAATGGGAATTAGCCATGGTCC		
SE006-*rfaZ*-red-F	GATAAAAATACGAGTTTTTTATGGGCAGCGTTAACTTCATGTAGGCTGGAGCTGCTTC	SE006, Δ*rfaZ*::kan^r	This study
SE006-*rfaZ*-red-R	TAGACAATTTTATCGTAATATATTTCATCCTCAAGTTCCGATGGGAATTAGCCATGGTCC		
SE006-*ompC*-red-F	ATAAGGCATGAAAAAAGGGCCCGCAG	SE006, Δ*ompC*::kan^r	This study

	GCCCTTTAGCAACATCTTTTGCTGATGT AGGCTGGAGCTGCTTC		
SE006-*ompC*-red-R	AAGCAGTGGCATAAAAAAGCAATAAAG GCATATAACAGAGGGTTAATAACATGG GAATTAGCCATGGTCC		
SE006-*btuB*-red-F	ATGATTAAAAAAGCTACGCTGCTGACG GCGTTCTCCGTCTGTAGGCTGGAGCT GCTTC	SE006, Δ*btuB*::kan^r	This study
SE006-*btuB*-red-R	TAATGGCGTATCGGTAATCGCATTACGC GCATCAACGTAAATGGGAATTAGCCATG GTCC		
SE006-*tolC*-red-F	TTTTTACAAATTGATCAGCGCTAAATAC TGCTTCACAACAAGGAATGCATGTAGG CTGGAGCTGCTTC	SE006, Δ*tolC*::kan^r	This study
SE006-*tolC*-red-R	AGACCTACAAGGGCACAGGTCTGATAA GCGCAGCGCCAGCGAATAACTTAATG GGAATTAGCCATGGTCC		
SE006-*flgK*-red-F	GTCCACGTAGTCGCTGCCGATAACAAC GAGTATTGAAGGATTAAAAGGAACCATC TGTGTAGGCTGGAGCTGCTTC	SE006, Δ*flgK*::kan^r	This study
SE006-*flgK*-red-R	CTCATATTTTGTTCGTACATCATCTGGG TACTGATACGCATGTCATCCTTCTCCTA TGGGAATTAGCCATGGTCC		
SE006-*fhuA*-red-F	AATAATAATTATCGTTTACGTTATCATT CACTTTCATCAGAGATATACCATGTGTA GGCTGGAGCTGCTTC	SE006, Δ*fhuA*:kan^r	This study
SE006-*fhuA*-red-R	GAAACGGAAGGTTGCCGTTGCAACGA CCTGACGTTCTGCGCCCCAGAAACATG GGAATTAGCCATGGTCC		
SE006-*lamB*-red-F	TTTTAGAAGGTGGCAGCGTTTAAAGAA AAGCAATGATCTTGTAGGCTGGAGCTG CTTC	SE006, Δ*lamB*::kan^r	This study
SE006-*lamB*-red-R	AGGGGCTTGCGCCCCTCGTTACGTCA GATGACCATCGTAATGGGAATTAGCCA TGGTCC		
SE006-*phoE*-red-F	GGGTTTCCCGACAAATCATAGCGCGTA ATTAAAACAGGATGTAGGCTGGAGCTG CTTC	SE006, Δ*phoE*::kan^r	This study
SE006-*phoE*-red-R	TGCCTGATGGCGCAGCGCCATCAGGC ACAATGCGACTTAATGGGAATTAGCCA TGGTCC		
SE006-*motA*-red-F	TCATGCTTCCTCAGTCGTCTGCTGCTG GTTTGGGTTTCTGTAGGCTGGAGCTGC TTC	SE006, Δ*motA*::kan^r	This study

SE006-*motA*-red-R	TACCTGGTGGTTATCGGTACAGTTTTCG GCGGTTATGTCATGGGAATTAGCCATG GTCC		
SE006-*ompA*-red-F	AAAAACCCCGCGACGCGGGGTTTTTTA TCAGACGGAAACTGTAGGCTGGAGCT GCTTC	SE006, Δ*ompA*::kan^r	This study
SE006-*ompA*-red-R	TATTCATGGCGTATTTTGGATGATAACG AGGCGCAAAAAATGGGAATTAGCCATG GTCC		
SE006-*ompF*-red-F	AGTCCTGTTTTTGAGGCATAAAACAAA GGGGTCTGCTGATGTAGGCTGGAGCT GCTTC	SE006, Δ*ompF* ::kan^r	This study
SE006-*ompF*-red-R	GCAGGTGTCATATAAAAAAACCAATGA GGGTAATAAATAATGGGAATTAGCCAT GGTCC		
SE006-*ompX*-red-F	TAAAACTTAGGACTTACTTGAAGCACAT TTGAGGTGGTTTGTAGGCTGGAGCTGC TTC	SE006, Δ*ompX*::kan^r	This study
SE006-*ompX*-red-R	CCGCCCCGAAAGGCGGATTTTTCATTT TCACCGACGTGAATGGGAATTAGCCAT GGTCC		
SE006-*tsX*-red-F	AAACGGGCCAGAGGACGACCTCTGGC CTTTTTTGCAGGTTGTAGGCTGGAGCT GCTTC	SE006, Δ*tsX*::kan^r	This study
SE006-*tsX*-red-R	TTTTCACTCCCGCAAGGGATTTCAAACA GTGGCATACATATGGGAATTAGCCATGG TCC		

Supplementary Table 4 Complementary strain primers used in this study.

**Primer**	**Nucleotide sequence [5`→ 3`]**	**Complementary strains**	**References**
pHSG396-*rfbP*-BamHI-F	CGGGATCCTCAGAGGGT GAGGATTAAATGGAT	SE006, Δ*rfbP*::kan^r (pHSG396-*rfbP*)	This study
pHSG396-*rfbP*-KpnI-R	GGGGTACCTTAATACGCA CCATCTCGCCG		
pHSG396-*rfaB*-BamHI-F	CGGGATCCATGAAAATAG CATTTATTGGCGAAGCG	SE006, Δ*rfaB*::kan^r (pHSG396-*rfaB*)	This study
pHSG396-*rfaB*-KpnI-R	GGGGTACCTCACTTTTGT AATTTCGAGAATATTGC		
pHSG396-*rfaC*-BamHI-F	CGGGATCCATGCGGGTTT TGATCGTTAAAAC	SE006, Δ*rfaC*::kan^r (pHSG396-*rfaC*)	This study
pHSG396-*rfaC*-EcoRI-R	CGGAATTCTTAATGAATCT TTCCAAATACGCTTGTG		
pHSG396-*rfaD*-BamHI-F	CGGGATCCATGATCATCG	SE006, Δ*rfaD*::kan^r (pHSG396-*rfaD*)	This study

	TTACCGGCG		
pHSG396-*rfaD*-EcoRI-R	CGGAATTCTTACGCGTCG CGGTTCAGC		
pHSG396-*rfaE*-BamHI-F	CGGGATCCATGAAAGTAA ATCTGCCAGCGT	SE006, Δ*rfaE*::kan^r (pHSG396-*rfaE*)	This study
pHSG396-*rfaE*-KpnI-R	GGGGTACCATGTCCGAC GCACCTTCAAAATC		
pHSG396-*rfaF*-BamHI-F	CGGGATCCATGAAAATTT TGGTCATTGGCCC	SE006, Δ*rfaF*::kan^r (pHSG396-*rfaF*)	This study
pHSG396-*rfaF*-EcoRI-R	CGGAATTCTTAAACGCCC TCTTCCGACAAC		
pHSG396-*rfaG*-BamHI-F	CGGGATCCATGAGAGTTG CCTTTTGCTTATATAAA	SE006, Δ*rfaG*::kan^r (pHSG396-*rfaG*)	This study
pHSG396-*rfaG*-EcoRI-R	CGGAATTCTCAACCATCT AAATCACCTGTAATAATAT CC		
pHSG396-*rfaH*-BamHI-F	CGGGATCCATGCAATCCT GGTATTTACTGTACT	SE006, Δ*rfaH*::kan^r (pHSG396-*rfaH*)	This study
pHSG396-*rfaH*-EcoRI-R	CGGAATTCAGGTCGTATT CTGAACGATAGC		
pHSG396-*rfaI*-BamHI-F	CGGGATCCATGAGCAGAA AATATTTTGAAGAAGAAG	SE006, Δ*rfaI*::kan^r (pHSG396-*rfaI*)	This study
pHSG396-*rfaI*-EcoRI-R	CGGAATTCTGAATCCATT ACATCACCTATGGG		
pHSG396-*rfaJ*-BamHI-F	CGGGATCCATGGATTCAT TTCCTGAGATAGAAAT	SE006, Δ*rfaJ*::kan^r (pHSG396-*rfaJ*)	This study
pHSG396-*rfaJ*-EcoRI-R	CGGAATTCATCATAATAG AGATTTAGGCAGGGGA		
pHSG396-*rfaK*-BamHI-F	CGGGATCCTGAGTTGCTT CGGCACGCAT	SE006, Δ*rfaK*::kan^r (pHSG396-*rfaK*)	This study
pHSG396-*rfaK*-EcoRI-R	CGGAATTCGTCCGGACTC AATCACTTATCAAAC		
pHSG396-*rfaL*-BamHI-F	CGGGATCCATGCTAACCA CATCATTAACGT	SE006, Δ*rfaL*:kan^r (pHSG396-*rfaL*)	This study
pHSG396-*rfaL*-EcoRI-R	CGGAATTCTTATCTATTTC TTAGCGCCAACAGAA		
pHSG396-*rfaP*-BamHI-F	CGGGATCCATGGTTGAGC TGAAAGCGC	SE006, Δ*rfaP*::kan^r (pHSG396-*rfaP*)	This study
pHSG396-*rfaP*-EcoRI-R	CGGAATTCCATTCTGTCT ATCCTTGTCTCACTC		
pHSG396-*rfaQ*-BamHI-F	CGGGATCCATGCGTTTTC ATGGAGACATGTT	SE006, Δ*rfaQ*::kan^r (pHSG396-*rfaQ*)	This study
pHSG396-*rfaQ*-EcoRI-R	CGGAATTCTCATAGCAGG CTGTCCAAATCTATG		

pHSG396-*rfaY*-BamHI-F	CGGGATCCTCCCCTGCCT AAATCTCTATT	SE006, Δ*rfaY*::kan^r (pHSG396-*rfaY*)	This study
pHSG396-*rfaY*-EcoRI-R	CGGAATTCTCAGCGCTTT TCCTTACCCTTAATA		
pHSG396-*rfaZ*-BamHI-F	CGGGATCCATGGGCAGC GTTAACTTCATAAC	SE006, Δ*rfaZ*::kan^r (pHSG396-*rfaZ*)	This study
pHSG396-*rfaZ*-EcoRI-R	CGGAATTCTAATGATGTAT CTAGTGTCTAGACAA		
pHSG396-*btuB*-BamHI-F	CGGGATCCATGAAGCCTG CGGCATCCTT	SE006, Δ*btuB*::kan^r (pHSG396-*btuB*)	This study
pHSG396-*btuB*-EcoRI-R	CGGAATTCTTAATGAATCT TTCCAAATACGCTTGTG		
pHSG396-*tolC*-BamHI-F	CGGGATCCATGCAAATGA AGAAATTGCTCCCCA	SE006, Δ*tolC*::kan^r(pHSG396-*tolC*)	This study
pHSG396-*tolC*-EcoRI-R	CGGAATTCTCAATGCCGG AATGGATTGCCGTTA		

Supplementary Table 5 Table shows all possible phage community compositions used in this study.

Combinations ID	Phage presence in the combinations (0: absence; 1: presence)				Number of phages
	GSP162	GSP193	GSP001	GSP032	
1	1	0	0	0	1
2	0	1	0	0	1
3	0	0	1	0	1
4	0	0	0	1	1
5	1	1	0	0	2
6	1	0	1	0	2
7	1	0	0	1	2
8	0	1	1	0	2
9	0	1	0	1	2
10	0	0	1	1	2
11	1	1	1	0	3
12	1	1	0	1	3
13	1	0	1	1	3
14	0	1	1	1	3
15	1	1	1	1	4

Supplementary Table 6 Antibiotic susceptibility of phage-resistant

strains (μg/mL).

strains	colistin	erythromycin	doxycycline	gentamicin	ceftiofur	meropenem	SDS
SE006	2	64	256	1	2	0.125	>512
GSP162-R1	0.5	64	256	1	2	0.125	>512
GSP162-R2	0.5	64	256	1	2	0.125	>512
GSP162-R3	1	64	256	1	2	0.125	>512
GSP162-R4	0.5	64	256	1	2	0.125	>512
GSP162-R5	0.5	64	256	1	2	0.125	>512
GSP162-R6	0.5	64	256	1	2	0.125	>512
GSP162-R7	0.5	64	256	0.5	2	0.125	>512
GSP162-R8	0.5	64	256	0.5	2	0.125	>512
GSP162-R9	1	64	256	1	2	0.125	>512
GSP162-R10	1	64	256	1	2	0.125	>512
GSP162-R11	1	64	256	0.5	2	0.125	>512
GSP162-R12	1	64	256	1	2	0.125	>512
GSP162-R13	1	64	256	1	2	0.125	>512
GSP162-R14	0.5	64	256	0.5	2	0.125	>512
GSP162-R15	0.5	64	256	0.5	2	0.125	>512
GSP162-R16	1	64	256	1	2	0.125	>512

strains	colistin	erythromycin	doxycycline	gentamicin	ceftiofur	meropenem	SDS
SE006	2	64	256	1	2	0.125	>512
GSP193-R1	0.25	2	64	0.5	1	0.125	128
GSP193-R2	0. 25	64	128	1	2	0.125	>512
GSP193-R3	0.0625	64	256	1	2	0.125	>512
GSP193-R4	0.5	64	128	1	2	0.125	>512
GSP193-R5	0.5	64	128	1	2	0.125	>512
GSP193-R6	0.25	2	64	0.5	1	0.125	128
GSP193-R7	0.25	64	128	1	2	0.125	>512
GSP193-R8	0.25	64	256	1	2	0.125	>512
GSP193-R9	0.5	64	256	1	2	0.125	>512
GSP193-R10	0.25	64	128	1	2	0.125	>512
GSP193-R11	0.25	2	64	0.5	1	0.125	128
GSP193-R12	0.5	64	256	1	2	0.125	>512
GSP193-R13	0.5	64	256	1	2	0.125	>512
GSP193-R14	0.25	64	64	0.5	2	0.125	>512
GSP193-R15	0.5	64	256	1	2	0.125	>512
GSP193-R16	0.25	64	64	0.5	2	0.125	>512

strains	colistin	erythromycin	doxycycline	gentamicin	ceftiofur	meropenem	SDS
SE006	2	64	256	1	2	0.125	>512
GSP001-R1	2	64	256	1	2	0.125	>512
GSP001-R2	2	64	256	1	2	0.125	>512

GSP001-R3	2	64	256	1	2	0.125	>512
GSP001-R4	2	64	256	1	2	0.125	>512
GSP001-R5	2	64	256	1	2	0.125	>512
GSP001-R6	2	64	256	1	2	0.125	>512
GSP001-R7	2	64	256	1	2	0.125	>512
GSP001-R8	2	64	256	1	2	0.125	>512
GSP001-R9	2	64	256	1	2	0.125	>512
GSP001-R10	2	64	256	1	2	0.125	>512
GSP001-R11	2	64	256	1	2	0.125	>512
GSP001-R12	2	64	256	1	2	0.125	>512
GSP001-R13	2	64	256	1	2	0.125	>512
GSP001-R14	2	64	256	1	2	0.125	>512
GSP001-R15	2	64	256	1	2	0.125	>512
GSP001-R16	2	64	256	1	2	0.125	>512

strains	colistin	erythromycin	doxycycline	gentamicin	ceftiofur	meropenem	SDS
SE006	2	64	256	1	2	0.125	>512
GSP032-R1	0.25	2	64	0.25	1	0.125	128
GSP032-R2	0.5	4	32	0.5	0.125	0.125	32
GSP032-R3	0.25	2	64	0.25	1	0.125	128
GSP032-R4	1	64	256	1	2	0.125	>512
GSP032-R5	0.5	4	32	0.5	0.125	0.125	32
GSP032-R6	0.5	4	32	0.5	0.125	0.125	128
GSP032-R7	1	64	256	1	2	0.125	>512
GSP032-R8	1	64	256	1	2	0.125	>512
GSP032-R9	1	64	256	1	2	0.125	>512
GSP032-R10	1	64	256	1	2	0.125	>512
GSP032-R11	0.5	4	32	0.5	0.125	0.125	32
GSP032-R12	0.25	2	64	0.25	1	0.125	128
GSP032-R13	0.25	2	64	0.25	1	0.125	128
GSP032-R14	0.5	4	32	0.5	0.125	0.125	32
GSP032-R15	0.5	4	32	0.5	0.125	0.125	32
GSP032-R16	1	64	256	1	2	0.125	>512

strains	colistin	erythromycin	doxycycline	gentamicin	ceftiofur	meropenem	SDS
SE006	2	64	256	1	2	0.125	>512
GSP-R1	0.25	2	64	0.25	1	0.0625	128
GSP-R2	0.25	2	64	0.25	1	0.0625	128
GSP-R3	0.0625	64	128	0.5	2	0.0625	>512
GSP-R4	0.25	2	64	0.25	0.25	0.0625	128
GSP-R5	0.25	2	64	0.5	1	0.03125	128
GSP-R6	0.25	2	64	0.25	1	0.0625	128
GSP-R7	0.25	2	64	0.25	1	0.0625	128
GSP-R8	0.0625	2	64	0.25	1	0.03125	128

GSP-R9	0.25	2	128	0.25	1	0.0625	>512
GSP-R10	0.25	4	64	0.25	1	0.0625	256
GSP-R11	0.25	2	128	0.25	1	0.0625	>512
GSP-R12	0.25	2	64	0.25	1	0.0625	128
GSP-R13	0.25	2	64	0.25	0.25	0.03125	128
GSP-R14	0.0625	64	128	0.5	2	0.0625	>512
GSP-R15	0.25	2	64	0.25	1	0.0625	128
GSP-R16	0.25	2	64	0.25	1	0.0625	128
